# Histone acetylation-dependent clustering of BRD2 instructs transcription dynamics

**DOI:** 10.1038/s41588-026-02533-x

**Published:** 2026-04-09

**Authors:** Niyazi Umut Erdogdu, Sukanya Guhathakurta, Ronald Oellers, Maria Shvedunova, Jose A. Morin, Eric M. Patrick, Janine Seyfferth, Ward Deboutte, Alejandro Gomez-Auli, Gerhard Mittler, Ibrahim I. Cissé, Asifa Akhtar

**Affiliations:** 1 Max Planck Institute of Immunobiology and Epigenetics, Freiburg, Germany; 2 Faculty of Biology, University of Freiburg, Freiburg, Germany; 3 CIBSS—Centre for Integrative Biological Signalling Studies, University of Freiburg, Freiburg, Germany

**Keywords:** Epigenetics, Gene regulation, Transcriptomics

## Abstract

Bromodomain (BD) and extra-terminal domain (BET) proteins are key regulators of RNA polymerase II (Pol II)-mediated transcription and their BDs represent promising drug targets. Yet, the interplay between histone acetylation and the chromatin dynamics of individual BET proteins with respect to transcriptional regulation is not fully understood. Here in mouse embryonic stem cells, we uncover an essential role of BRD2 in maintaining Pol II recruitment at promoters through its interaction with TFIID, which becomes particularly critical under the conditions of impaired pause release. Combining rapid protein degradation, chemogenomics and super-resolution microscopy, we show that MOF-mediated histone H4 acetylation promotes BRD2 chromatin association, which in turn enables BRD2 clustering. Accordingly, MOF depletion or deletion of the BRD2’s intrinsically disordered region largely recapitulates defects in promoter enrichment and clustering of the transcription machinery observed upon BRD2 loss. Thus, these findings support a model in which histone acetylation-dependent spatiotemporal dynamics of BRD2 coordinate the transcription machinery to regulate transcription initiation.

## Abstract (web summary)

BRD2 is key for RNA polymerase II recruitment at promoters, becoming critical in the absence of BRD4 or upon pause-release inhibition. Depletion of MOF or deletion of BRD2’s intrinsically disordered region mimics transcriptional defects caused by BRD2 loss.

## Main

Bromodomain (BD) and extra-terminal (ET) domain (BET) proteins BRD2, BRD3, BRD4 and the testis-specific BRDT are transcriptional regulators characterized by their signature structural organization as two N-terminal tandem BDs followed by an ET domain^1^. Over the years, they have gained significant attention, as they are pivotal activators of transcriptional networks related to cancer and inflammation^2^. The development of BET inhibitors, which disrupt the BD-dependent chromatin binding of BET proteins, provided a promising tool for the suppression of oncogenic pathways^3,4^.

Despite extensive research on these proteins in transcription regulation, a clear mechanistic consensus has only emerged for BRD4. BRD4 promotes the transition of proximally paused RNA polymerase II (Pol II) into productive elongation by forming a catalytically active BRD4-pTEFb and facilitating the formation of functional elongation complexes^5–8^. In contrast, the transcriptional roles of BRD2 and BRD3 remain less well-defined. It has been previously shown that both BRD2 and BRD3 enable Pol II to transcribe through nucleosomes containing specific histone H4 modifications such as H4K5ac and H4K12ac in a defined in vitro chromatin transcription system^9^. However, their rapid depletion in cells did not lead to any major changes in global transcription elongation, suggesting that these proteins execute more nuanced regulatory functions in transcription^10,11^.

## Results

### MOF-mediated H4 acetylation (H4ac) is required for BRD2 recruitment to chromatin

**Fig. 1:**
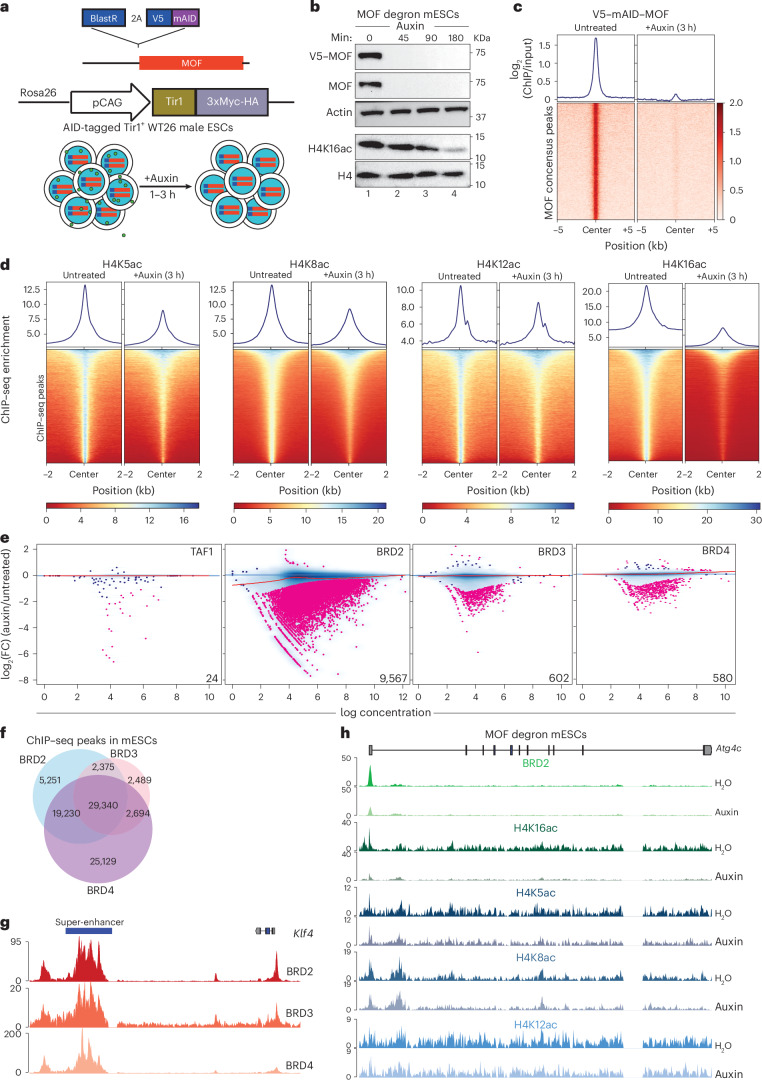
MOF-mediated H4ac is required for the recruitment of BRD2 to the chromatin. **a**, Schematic showing the genome editing strategy to generate an AID line for MOF. **b**, WB showing rapid degradation of MOF in MOF degron mESCs upon auxin treatment for indicated time points. Actin serves as a loading control. **c**, Metagene plot and heatmap of input-normalized ChIP–seq signal for MOF from MOF degron mESCs upon auxin treatment for 3 h (*n* = 2). The signal was plotted over MOF consensus peaks and MOF was immunoprecipitated using V5 antibody. **d**, Metagene plot and heatmap of spike-in-normalized native ChIP–seq signal for H4K5ac, H4K8ac, H4K12ac and H4K16ac over their respective peaks upon auxin treatment of MOF degron mESCs for 3 h (*n* = 3). **e**, MA plots of differential chromatin enrichment analysis for TAF1, BRD2, BRD3 and BRD4 upon auxin treatment of MOF degron mESCs for 3 h (*n* = 2–3). Pink dots depict differentially enriched peaks with FDR < 0.05. **f**, Venn diagram depicting the distinct and overlapping peaks of BRD2, BRD3 and BRD4 ChIP–seq peaks in mESCs. **g**, Example genome snapshot of BRD2, BRD3 and BRD4 ChIP–seq signals over the super-enhancer region near the *Klf4* gene. **h**, Genome snapshot of the MOF-sensitive BRD2 target gene *Atg4c* showing H4K5ac, H4K8ac, H4K12ac, H4K16ac and BRD2 ChIP–seq signals upon auxin treatment of MOF degron mESCs for 3 h. AID, auxin-inducible degron; WB, western blot; FDR, false discovery rate. Source data

**Extended Data Fig. 1:**
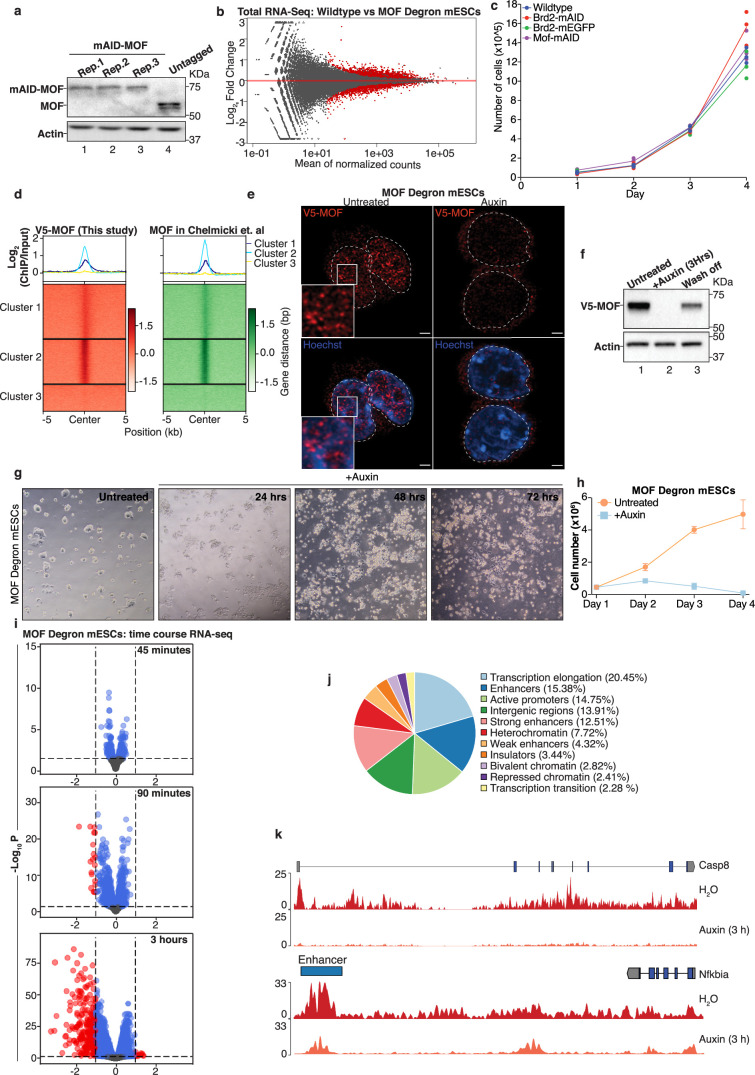
Characterization of MOF degron mESCs. **a**, Immunoblot analysis of untagged and mAID-tagged MOF mESCs using antibody against MOF. Actin served as a loading control. **b**, MA plot depicting the log_2_ fold changes of total RNA levels in relation to mean of normalized counts from parental WTA7 and MOF degron mESCs (n = 3). Red dots depict all the differentially expressed genes with abs(log_2_(FC)) > 0 & p-adj< 0.05. DEGs with abs(log_2_(FC)) > 1: 25, abs(log_2_(FC)) > 0.5: 121. **c**, Growth curve analysis of wild-type, mAID-MOF, BRD2-mAID and BRD2-mEGFP mESCs. *n* = 3 independent experiments. **d**, Metagene plots and heatmaps showing input-normalized ChIP–seq enrichment of V5–MOF (generated in this manuscript using antibody against V5) and untagged MOF (generated in ref. ^1^ using an antibody against MOF) in male mESCs. MOF consensus peaks were sorted into 3 clusters by k-means clustering. **e**, Immunofluorescence microscopy images of untreated and auxin-treated (12 h) MOF degron mESCs using an antibody against the V5-tag (red) and nuclear Hoechst staining (blue). The dashed line represents the nuclear boundary. Scale bar of images: 5 μm. **f**, Immunoblot analysis of MOF degron mESCs left untreated, treated with auxin for 3 h (‘+auxin (3 h)’) or treated with auxin for 3 h followed by wash-off of auxin and incubation in auxin-free media for 12 h (‘wash off’). **g**, Phase contrast microscopy images of untreated and auxin-treated MOF degron mESCs to qualitatively monitor cellular morphology and colony-forming efficiency. **h**, Growth curve of untreated and auxin-treated MOF degron mESCs. Data represent mean ± s.e.m., *n* = 3 independent experiments. **i**, Time-course RNA-seq analyses of MOF degron mESCs (*n* = 3 independent experiments) upon auxin treatment. Volcano plots show all expressed transcripts with horizontal dashed line indicating P_adj_ < 0.05 significance cutoff and vertical dashed line indicating the log_2_ fold change cutoff (log_2_(FC) < −1 & log_2_(FC) > 1). DESeq analysis was performed taking untreated cells as reference for all the conditions. Statistical significance was determined using a Wald test implemented in DESeq2, followed by correction for multiple comparisons using the Benjamini–Hochberg method. **j**, Pie chart depicting the percentage distribution of H4K16ac peaks with different chromatin features obtained from chromHMM analysis. **k**, Example genome snapshots depicting the enrichment of H4K16ac ChIP–seq signal over promoters and gene bodies (top) and enhancers (bottom). Source data

Histone H4ac has been shown to recruit BET proteins to chromatin through their BDs in vitro^12^. To investigate the role of H4ac in the control of transcription dynamics, we decided to deplete the MYST family lysine acetyltransferase KAT8/MOF, which primarily catalyzes H4K16ac in vivo and has been shown to additionally acetylate H4K5/H4K8/H4K12 in vitro^13,14^. To study the direct consequences of MOF depletion, we generated a rapid degradation system by endogenously tagging MOF with V5 and mAID tags in mouse embryonic stem cells (mESCs) that stably express the E3 Ub ligase Tir1 from *Oryza sativa* from a cassette introduced into the *Rosa26* locus (Tir1^+^ WT26 mESCs; Fig. 1a). The V5–mAID tag did not markedly affect MOF chromatin binding, cell growth or the transcriptome (Extended Data Fig. 1a–d)^15^.

Auxin treatment resulted in near-complete, reversible degradation of MOF within 45 min (Fig. 1b and Extended Data Fig. 1e,f). Bulk H4K16ac levels decreased only after 3 h of auxin treatment, indicating that the turnover of this histone mark requires at least a few hours in the absence of new catalysis (Fig. 1b). Chromatin immunoprecipitation followed by sequencing (ChIP–seq) validated loss of MOF occupancy at its binding sites upon auxin treatment (Fig. 1c). As previously reported, MOF depletion led to proliferation defects and cell death (Extended Data Fig. 1g,h)^16,17^.

We then performed a time-course total RNA-seq analysis to identify the earliest time point of auxin treatment at which a robust, direct transcriptional response to MOF depletion was elicited, without secondary effects. DESeq analysis revealed an increase in differentially expressed genes with increasing treatment time. Because both the first robust transcriptional response and the onset of bulk H4K16ac loss occurred at 3 h of auxin treatment, we chose this time point for subsequent experiments (Fig. 1b and Extended Data Fig. 1i).

**Extended Data Fig. 2:**
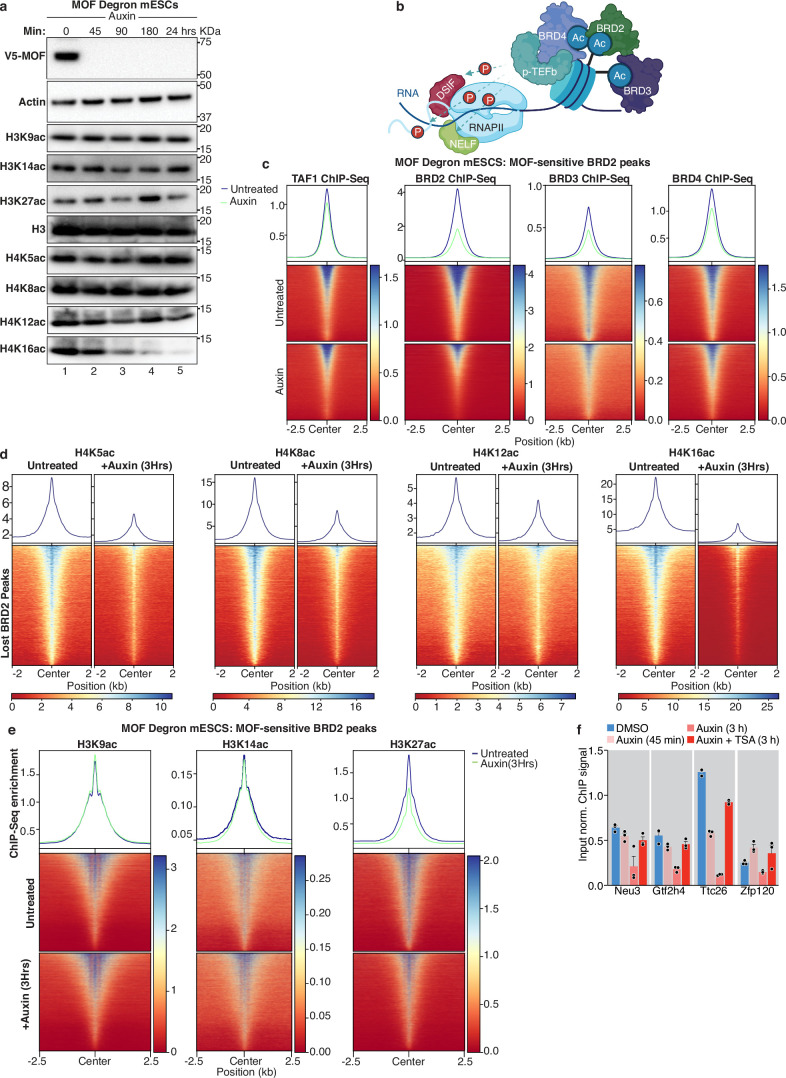
Characterization of histone acetylation response upon acute MOF depletion. **a**, Immunoblot analyses of bulk histone H3 and H4 modifications at different time points of auxin-treated MOF degron mESCs. Actin and total histone H3 served as a loading control. **b**, Schematic depicting the enrichment of BET BRD members BRD2, BRD3 and BRD4 on acetylated chromatin. Schematic in **b** created in BioRender; Erdogdu, U. https://biorender.com/8t7cth9 (2026). **c**, Metagene plots and heatmaps showing ChIP–seq enrichment of TAF1 and BET proteins in MOF degron mESCs upon auxin treatment for 3 h. **d**,**e**, Metagene plots and heatmaps of spike-in-normalized native ChIP–seq signal for histone H4ac (H4K5ac, H4K8ac, H4K12ac and H4K16ac) (**d**) and histone H3ac (H3K9ac, H3K14ac and H3K27ac) (**e**) over MOF-sensitive BRD2 peaks (Fig. 1e) in MOF degron mESCs upon auxin treatment for 3 h. The signal was plotted over BRD2 ChIP–seq peaks. **f**, BRD2 ChIP–qPCR in MOF degron mESCs upon auxin treatment for 45 min, 3 h and cotreatment with histone deacetylase inhibitor TSA. ChIP enrichment for selected MOF targets was normalized to their inputs. Data indicate mean ± s.e.m., n = 3 biological replicates. Source data

To investigate the direct contribution of MOF to H4ac, we performed native ChIP–seq for H4ac marks upon MOF depletion. Loss of MOF led to overall reduced H4K5/H4K8/H4K12/H4K16ac levels, with the most severe effect on H4K16ac (Fig. 1d). Furthermore, 65% of all H4K16ac ChIP–seq peaks were associated with chromatin features related to active transcription, such as transcription elongation, active promoters and enhancers (Extended Data Fig. 1j,k). By contrast, MOF depletion did not significantly affect H3K9/H3K14/H3K27ac levels (Extended Data Fig. 2a).

The prevailing view is that histone acetylation can affect transcription through the binding of BD proteins, which recruit transcriptional cofactors that modulate Pol II activity^18^. In particular, H4ac has been implicated in BET protein recruitment to chromatin (Extended Data Fig. 2b)^9,12,19,20^. Additionally, the TAF1 subunit of the general transcription factor TFIID has been shown to bind H4ac peptides and has been proposed to be recruited to transcription start sites by MOF-mediated H4ac^21,22^. Differential chromatin occupancy analysis revealed minor changes in TAF1, BRD3 and BRD4 binding after MOF depletion (Fig. 1e). However, BRD2 chromatin occupancy was severely altered, with 9,567 binding sites showing loss of enrichment (Fig. 1e). This specific effect on BRD2 chromatin association was observed despite significant overlap in genome-wide BET protein binding sites in mESCs (Fig. 1f,g, Extended Data Fig. 2c and Supplementary Note 1).

We observed a reduction of H4K5ac, H4K8ac, H4K16ac and, to a lesser extent, H3K27ac and H4K12ac on MOF-sensitive BRD2 peaks, while H3K9ac and H3K14ac remained unchanged (Fig. 1h and Extended Data Fig. 2a,d,e). ChIP–qPCR showed that loss of BRD2 binding resembled the kinetics of H4K16ac depletion and was prevented by histone deacetylase inhibition with trichostatin A (Fig. 1b and Extended Data Fig. 2f). These findings demonstrate the important role of MOF-mediated H4ac in the locus-specific recruitment of BRD2 to chromatin.

### BET protein codepletion identifies their distinct roles in the regulation of nascent transcription

**Fig. 2:**
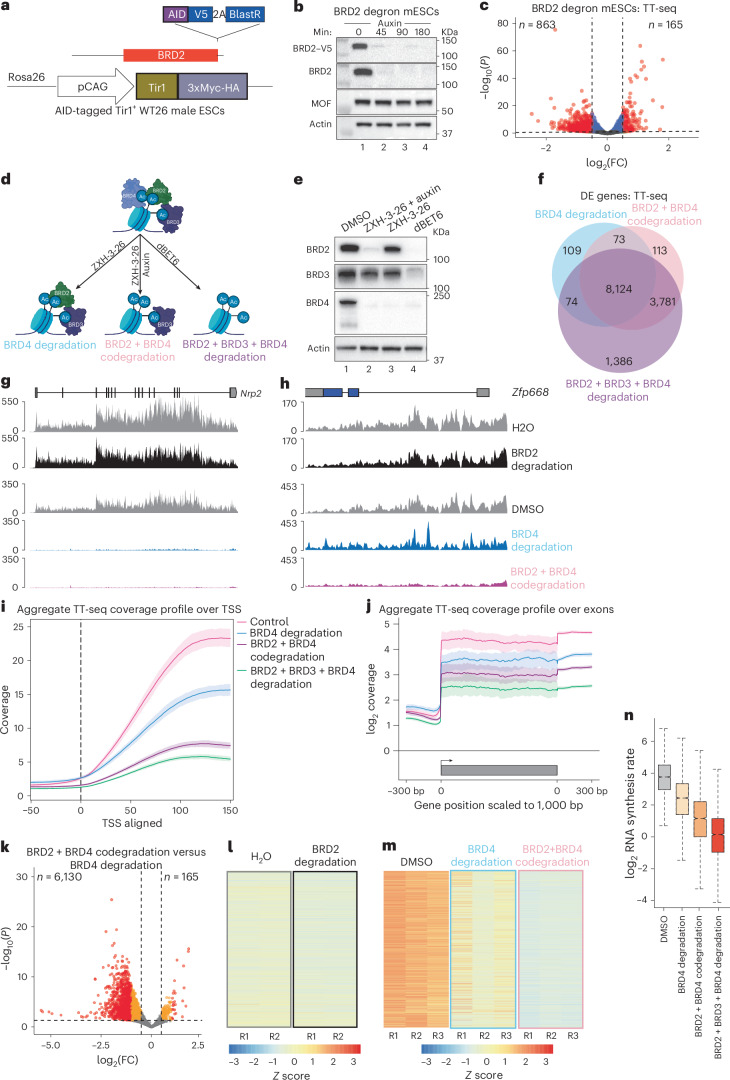
Acute codepletion of BET proteins identifies their distinct roles in promoting RNA synthesis. **a**, Schematic showing the genome editing strategy to generate an AID line for BRD2. **b**, WB showing the rapid degradation of BRD2 in BRD2 degron mESCs upon auxin treatment for different time points. Actin serves as a loading control. **c**, Volcano plot showing differentially expressed genes (red) identified by TT-seq from BRD2 degron mESCs treated for 3 h with auxin. The dashed lines mark the log_2_(FC) cutoff of 0.5 and adjusted *P*-value cutoff of 0.05 (*n* = 2). Statistical significance was determined using a Wald test implemented in DESeq2, followed by correction for multiple comparisons using the Benjamini–Hochberg method. **d**, Schematic depicting the experimental strategy for codepletion of BET BRD proteins. Schematic in **d** created in BioRender; Erdogdu, U. https://biorender.com/gwr80tg (2026). **e**, WB showing rapid degradation of BET BRD proteins upon combined treatments as described in **d**. **f**, Venn diagram showing the overlap of differentially expressed genes (log_2_(FC) < −1 and *P*_adj_ < 0.05) detected in TT-seq experiments upon codepletion of BET BRD proteins (*n* = 3). **g**,**h**, Genome snapshots of nascent RNA signals for representative genes *Nrp2* (**g**) and *Zfp668* (**h**) upon auxin treatment of BRD2 degron mESCs (top two tracks) and combined depletion of BRD2 and BRD4 (bottom three tracks). **i**,**j**, Aggregate plots depicting TT-seq coverage over TSSs (**i**) and exons (**j**) upon individual or combined BET BRD depletion. The solid lines show the trimmed mean coverage profile across genes, while the shaded area reflects the minimum–maximum range of coverage values obtained from bootstrap resampling. **k**, Volcano plot showing differentially expressed genes identified by TT-seq (red) upon codepletion of BRD2 and BRD4 in BRD2 degron mESCs (genes deregulated upon BRD2 and BRD4 codepletion compared to BRD4 depletion alone). The dashed lines mark the log_2_(FC) cutoff of 0.5 (vertical lines) and adjusted *P-*value cutoff of 0.05 (horizontal line). Red, transcripts with |log_2_(FC)| ≥ 1; orange, transcripts with 0.5 ≤ |log_2_(FC)| < 1. Statistical significance was determined using a Wald test implemented in DESeq2, followed by correction for multiple comparisons using the Benjamini–Hochberg method. **l**,**m**, Box plots depicting the *z* scores for nascent RNA levels of the significantly downregulated genes scored in **k** upon depletion of BRD2 alone (**l**) or upon codepletion of BRD2 and BRD4 (**m**). **n**, Box plot depicting the log_2_(synthesis rates) of the differential genes from **k** upon BET BRD depletions (*n* = 3,464 genes). The box plot represents the IQR, spanning from the first quartile to the third quartile, with the median shown as a line inside the box. The whiskers extend to the most extreme data points within 1.5× IQR. IQR, interquartile range; TSS, transcription start site. Source data

**Extended Data Fig. 3:**
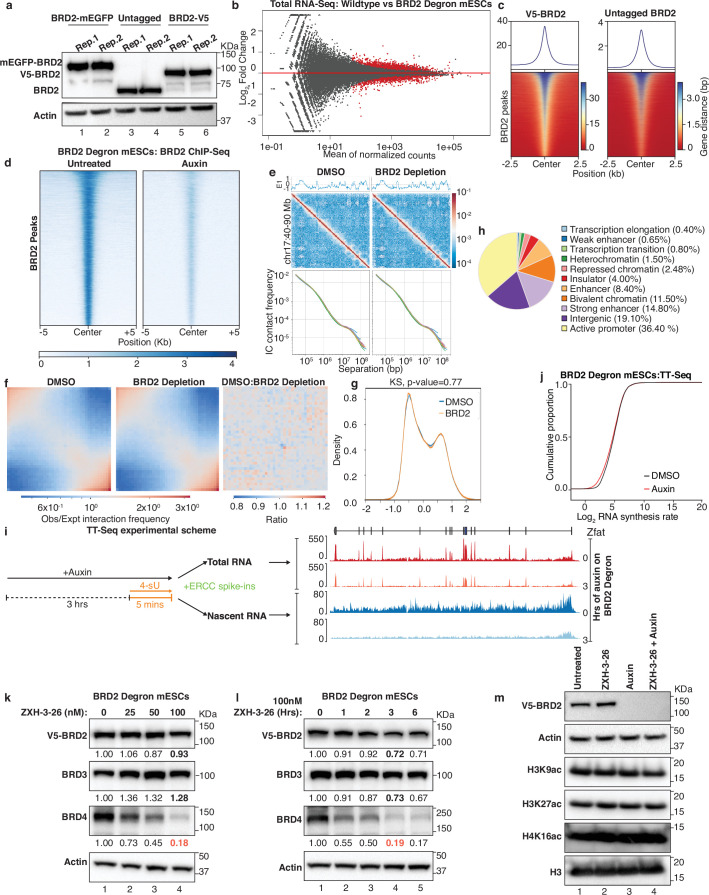
Characterization of BRD2 degron mESCs. **a**, Immunoblot analysis of untagged, mGFP and mAID-tagged BRD2 mESCs using antibody against BRD2. Actin served as a loading control. **b**, MA plot depicting the log_2_ fold changes of total RNA levels in relation to mean of normalized counts from WTA7 and BRD2 degron mESCs. Red dots depict all the differentially expressed genes with abs(log_2_(FC)) > 0 & p-adj< 0.05. DEGs with abs(log_2_(FC)) > 1: 9, abs(log_2_(FC)) > 0.5: 51. **c**, Metagene plots and heatmaps showing ChIP–seq enrichment of BRD2–V5 (generated using antibody against V5) and untagged BRD2 (generated using an antibody against BRD2) in mESCs. **d**, Heatmap depicting the input-normalized ChIP–seq signal of BRD2 in BRD2 degron mESCs upon 3 h auxin treatment. The signal was plotted over BRD2 peaks and the chromatin IP of BRD2 was performed using V5 antibody. **e**, Pearson’s correlation matrix of chromosome 17: 40–90 Mb upon acute BRD2 depletion in comparison to untreated control from micro-C experiment. **f**, Differential saddle plot analysis by micro-C upon acute BRD2 depletion for 3 h. **g**, Histogram of eigenvector values from Pearson’s correlation matrix upon BRD2 depletion for 3 h in comparison to untreated control from micro-C experiment. P-value was calculated using a two-sample Kolmogorov–Smirnov test. **h**, Pie chart depicting the genome-wide distribution of MOF-sensitive BRD2 peaks sorted by chromatin features obtained from chromHMM analysis. **i**, Schematics depicting the experimental strategy for TT-seq experiments (left). Genome snapshots of total RNA-seq and TT-seq signals over an example gene (*Zfat*) upon auxin treatment of BRD2 degron mESCs (right). **j**, ECDF plot of global RNA synthesis rates from control and BRD2-depleted mESCs. **k**,**l**, Immunoblot analysis of ZXH-3-26-treated BRD2 degron mESCs at different concentrations (**k**) and time points (**l**). Actin served as a loading control and was used to normalize signals from each BRD protein. Ratio of signal from each lane is indicated below and represented relative to the respective untreated condition. **m**, Immunoblot analysis of bulk histone modifications upon individual or codepletion of BRD2 and BRD4. Actin and total histone H3 served as loading controls. Source data

BRD2 has been implicated in the regulation of 3D genome organization and transcription dynamics^10,23,24^. To investigate the functional significance and direct role of the H4ac–BRD2 relationship in these processes, we generated an auxin-inducible BRD2 degron by endogenous mAID–V5 tagging of BRD2 in Tir1^+^ WT26 mESCs (Fig. 2a). The tag did not affect BRD2 protein levels, chromatin binding, cell growth or the transcriptome (Extended Data Figs. 1c and 3a–c). BRD2 was significantly depleted within 45 min of auxin treatment (Fig. 2b). Loss of BRD2 ChIP–seq peaks upon auxin treatment further demonstrated the robustness of the degron system (Extended Data Fig. 3d).

Acute BRD2 depletion for 3 h did not lead to significant changes in genome compartmentalization or topologically associating domain structures (Extended Data Fig. 3e–g and Supplementary Note 2). Because MOF-sensitive BRD2 peaks largely overlap with active chromatin regions (Extended Data Fig. 3h), we focused on the role of BRD2 in regulating transcription dynamics.

We performed transient transcriptome sequencing (TT-seq) in BRD2 degron mESCs to quantitatively interrogate the function of BRD2 in RNA synthesis (Extended Data Fig. 3i)^25^. As previously reported^10,11,26^, rapid BRD2 depletion did not affect RNA synthesis globally, but rather led to locus-specific, mild transcriptional changes (Fig. 2c and Extended Data Fig. 3j).

Because BET proteins frequently co-occupy the same chromatin binding sites, we suggested that they might have distinct, but collaborative roles in regulating nascent transcription. Therefore, we performed TT-seq upon BET protein codepletion to unravel their functional organization in transcription regulation. We combined auxin treatment of BRD2 degron mESCs with the BRD4-specific PROTAC ZXH-3-26 to codeplete BRD2 and BRD4, or with dBET6 to degrade all three BET proteins (Fig. 2d)^5,27^. Treatment with 100 nM of ZXH-3-26 efficiently depleted BRD4 within 3 h, while modestly affecting BRD2 and BRD3 to variable degrees (Fig. 2e and Extended Data Fig. 3k,l). We confirmed the efficacy of combined treatments in degrading target BET proteins (Fig. 2e). Individual or combined BRD2 and BRD4 depletions did not affect bulk H3K9, H3K27 or H4K16 acetylation (Extended Data Fig. 3m).

**Extended Data Fig. 4:**
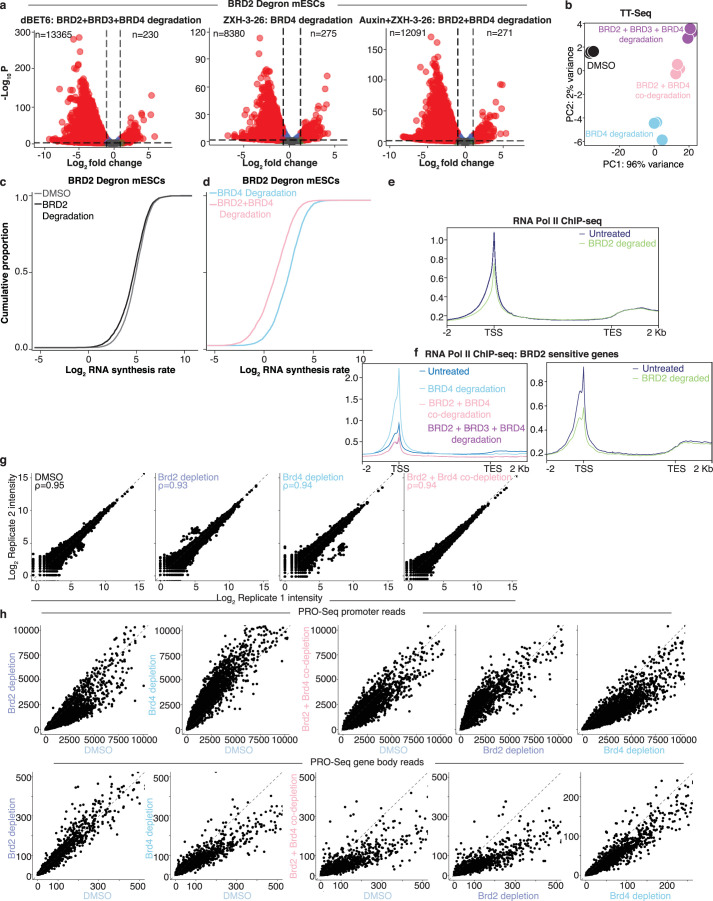
The distinct roles of BET BRD proteins in promoting nascent RNA synthesis. **a**, Volcano plots showing all expressed transcripts in the indicated condition with horizontal dashed line indicating P_adj_<0.05 significance cutoff and vertical dashed line indicating the log2 fold-change cutoff (abs(log_2_(FC)) > 1). DESeq analysis was performed taking DMSO-treated cells as reference for all the conditions. Statistical significance was determined using a Wald test implemented in DESeq2, followed by correction for multiple comparisons using the Benjamini–Hochberg method. **b**, PCA of TT-seq experiment upon BET protein codepletion depicting the clustering of the samples based on their treatment. **c**,**d**, ECDF plots showing the impact of BRD2 depletion on RNA synthesis rates for the differentially expressed genes from Fig. 2k in the presence (**c**) or absence (**d**) of BRD4. **e**, Metagene plots of RNA Pol II ChIP–seq upon acute BRD2 depletion for 3 h (n = 2). **f**, Metagene plots of RNA Pol II ChIP–seq upon sequential BET protein depletion (left) or acute BRD2 depletion alone (right) for 3 h over the differential genes in Fig. 2k. **g**, Scatterplots showing correlation between PRO-seq replicates (n = 2) over the promoters of expressed mRNAs. Spearman correlation values are indicated. **h**, Scatterplots depicting the changes of PRO-seq signal intensity between the indicated conditions over promoters (top) and gene bodies (bottom) of all expressed mRNAs.

Depletion of BRD4 or all BET proteins caused a severe defect in RNA synthesis, with 8,124 significantly downregulated transcripts that are common between the two conditions (log_2_ fold change (FC) < −1 and adjusted *P* value (*P*_adj_) < 0.05; Fig. 2f and Extended Data Fig. 4a). Additional 3,781 transcripts were significantly downregulated only upon the codepletion of BRD2 and BRD4 or all three BET proteins, compared to BRD4 depletion alone (Fig. 2f–h). A total of 1,386 transcripts were significantly downregulated only upon depletion of all three BET proteins, suggesting a nonredundant role of BRD3 in promoting RNA synthesis at a subset of genes (Fig. 2f). Aggregate TT-seq coverage profiles demonstrated progressive impairment of RNA synthesis with increasing numbers of BET proteins being degraded, with the strongest effects upon dBET6 treatment (Fig. 2i,j).

Principal component analysis revealed that codepletion of BRD2 and BRD4 resulted in transcriptional profiles closer to triple BET depletion than BRD4 depletion alone (Extended Data Fig. 4b). DESeq comparison identified 6,130 transcripts that were more severely downregulated (log_2_(FC) < −0.5 and *P*_adj_ < 0.05) upon codepletion of BRD2 and BRD4 than upon BRD4 degradation alone (Fig. 2k). We also found that the presence of BRD2 becomes more crucial for sustaining RNA synthesis for these 6,130 transcripts in the absence of BRD4, as revealed by the increased magnitude of transcriptional downregulation upon BRD2 loss in cells lacking BRD4 compared to cells retaining BRD4 (Fig. 2l,m). Transcriptional rate calculations^28^ showed that the impact of BRD2 loss on RNA synthesis rates for these transcripts was more severe in the absence of BRD4 (Fig. 2n and Extended Data Fig. 4c,d). These findings indicate that BRD2 and very likely also BRD3 provide a support function to prevent a transcriptional collapse in the absence of BRD4.

### BRD2 maintains Pol II occupancy at the promoters in the absence of BRD4

**Fig. 3:**
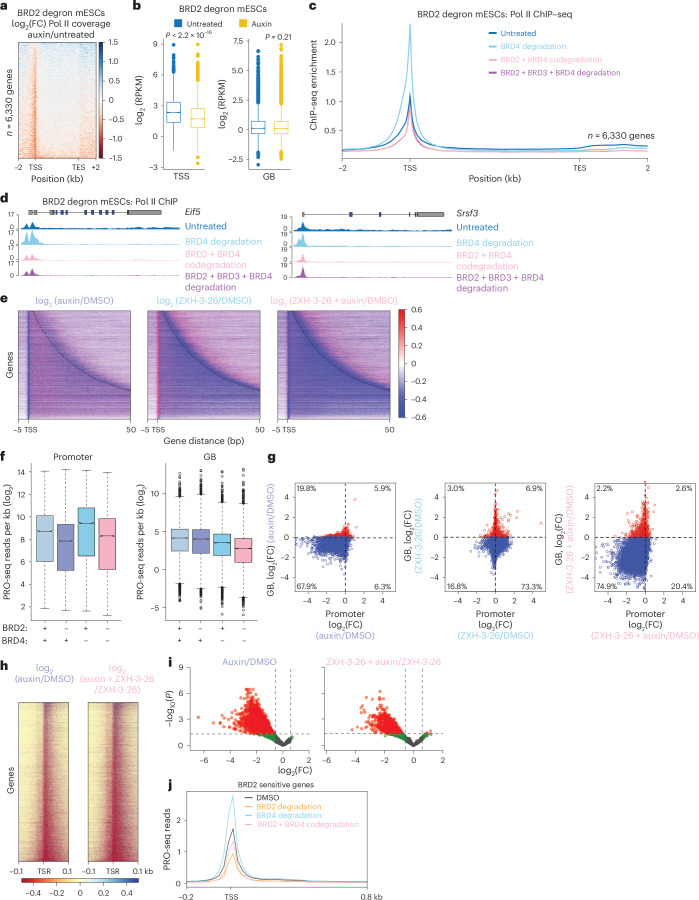
BRD2 regulates the chromatin occupancy of RNA Pol II at the promoters. **a**, ChIP–seq analysis depicting the log_2_(FC) of RNA Pol II coverage upon auxin treatment of BRD2 degron mESCs for 3 h (*n* = 2). **b**, Box plots depicting RPKM-normalized RNA Pol II coverages over TSSs and GBs upon auxin treatment of BRD2 degron mESCs for 3 h (*n* = 7,092 genes). **c**, Metagene plot showing RNA Pol II ChIP–seq signal upon codepletion of BET BRD proteins in BRD2 degron mESCs (*n* = 2). **d**, Genome snapshots depicting the changes in RNA Pol II ChIP–seq signal on the representative genes *Eif5* (left) and *Srsf3* (right). **e**, PRO-seq analysis depicting log_2_(FC) of RNA Pol II coverage upon individual or combined depletion of BRD2 and BRD4 in comparison to DMSO control over all expressed mRNAs (*n* = 2). **f**, Box plots depicting log_2_-scaled PRO-seq read density over the promoters and GBs upon BET BRD depletion (*n* = 10,356 genes). **g**, Scatterplots depicting the log_2_(FC) of PRO-seq signal at the promoters in comparison to that at the GBs from BRD2 degron mESCs treated as mentioned. The blue circles indicate reduced PRO-seq occupancy over the GBs and the red circles indicate increased PRO-seq occupancy over the GBs. **h**, Heatmap depicting the log_2_(FC) of 5′ PRO-seq reads over 11-bp TSRs upon auxin and auxin + ZXH-3-26 treatments in comparison to DMSO control and ZXH-3-26 treatment, respectively, in BRD2 degron mESCs. **i**, Volcano plots depicting log_2_(FC) of 5′ PRO-seq reads over TSRs upon auxin (left) and auxin + ZXH-3-26 (right) treatment in comparison to DMSO control and ZXH-3-26 treatment, respectively, in BRD2 degron mESCs. **j**, Metagene plot depicting the average PRO-seq read density over the promoters of the differential genes in Fig. 2k. The box plots in **b** and **f** represent the IQR, spanning from the first quartile to the third quartile, with the median shown as a line inside the box. The whiskers extend to the most extreme data points within 1.5× IQR. RPKM, reads per kilobase million; TES, transcription end site.

To dissect the functional divergence of BET proteins in transcription regulation, we performed Pol II ChIP–seq upon single or combined BET protein depletion. In line with transcriptional profiles, BRD2 depletion did not severely affect gene body (GB) Pol II occupancy while leading to a mild, but significant decrease at the promoters (Fig. 3a,b and Extended Data Fig. 4e). As reported^5,10^, a reduced RNA synthesis upon BRD4 depletion was accompanied by an increased Pol II enrichment at the promoters, reminiscent of a Pol II pause-release defect (Fig. 3c,d). Upon codepletion of all three BET proteins, promoter Pol II occupancy did not phenocopy BRD4 depletion and was rather comparable to dimethyl sulfoxide (DMSO) controls. Codepletion of BRD2 with BRD4 was sufficient to reverse the accumulation of promoter-proximally paused Pol II induced by BRD4 depletion (Fig. 3c,d). Similar Pol II ChIP–seq profiles were observed in these genes with more severe reduction in their RNA synthesis upon codepletion of BRD2 and BRD4 (Fig. 2k and Extended Data Fig. 4f).

We performed precision run-on sequencing (PRO-seq) upon BRD2 and BRD4 depletions to monitor catalytically engaged Pol II at single-nucleotide resolution (Extended Data Fig. 4g)^29^. Consistent with ChIP–seq and TT-seq, BRD4 depletion globally increased promoter-proximal Pol II while reducing its GB occupancy (Fig. 3e,f and Extended Data Fig. 4h). In contrast, BRD2 depletion reduced promoter Pol II occupancy and caused a moderate GB decrease (Fig. 3e–g and Extended Data Fig. 4h). Codepletion of BRD2 and BRD4 markedly reduced both promoter and GB Pol II levels (Fig. 3e–g and Extended Data Fig. 4h).

**Extended Data Fig. 5:**
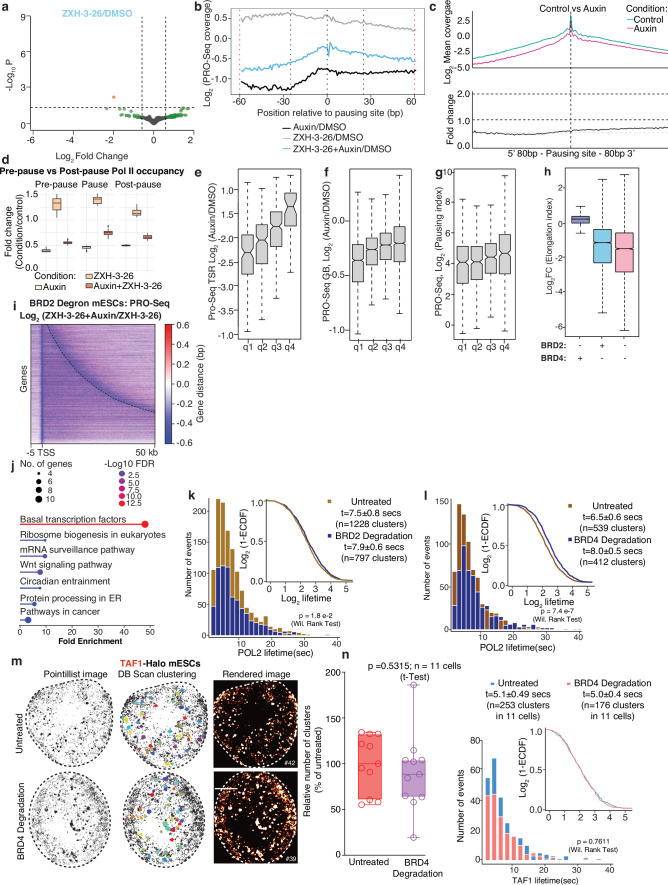
BRD2 regulates the dynamics of RNA Pol II and TAF1 clusters. **a**, Volcano plot depicting log_2_ fold changes and adjusted p-values of 5′ PRO-seq reads over TSRs upon ZXH-3-26 treatment in comparison to DMSO control in BRD2 degron mESCs. Statistical significance was determined using a Wald test implemented in DESeq2, followed by correction for multiple comparisons using the Benjamini–Hochberg method. **b**, Lineplot depicting the average changes in RNA Pol II occupancy at the promoters of all expressed genes at single-basepair resolution. log_2_ fold change of PRO-seq coverage was calculated for the indicated conditions. Prepause window was defined from −60bp to −25bp relative to the pausing site. **c**, Line diagram of mean PRO-seq 3′ read coverage in log_2_ scale over the pausing site ±80 bp from B2 degron mESCs upon auxin treatment. The line diagram below depicts log_2_ fold change (auxin/control) of the signal over the plotted region. **d**, Box plot depicting the log_2_ fold changes of PRO-seq 3′ reads over prepause (from pause_site −80 bp to −26 bp), pause (pause_site ±25 bp) and postpause (from pause_site +26 bp to pause site +80 bp) windows from BRD2 degron mESCs upon mentioned treatments in comparison to DMSO control (n = 8426 promoters). **e**, Box plot depicting log_2_ fold changes of PRO-seq 5′ reads over active mRNA promoters from BRD2 degron mESCs upon auxin treatment compared to the control. The data were divided into quartiles (q1–q4) based on log_2_ fold change (auxin/control) of TT-seq differentially expressed genes (p-adj< 0.05) (n = 744 genes). **f**, Box plot depicting log_2_ fold changes of PRO-seq gene body (GB) Pol II occupancy upon BRD2 degradation in degron mESCs. The data were divided into quartiles (q1–q4) based on log_2_ fold change (auxin/control) of 5′ PRO-seq reads over TSRs (n = 6148 genes). **g**, Box plot depicting log_2_ scaled pausing indices of actively transcribed genes under control conditions calculated from PRO-seq 3′ reads. The data were divided into quartiles of (q1–q4) based on log_2_ fold change (auxin/control) of TT-seq differentially expressed genes (p-adj < 0.05) (n = 1766 genes). **h**, Box plots depicting the log_2_ fold change of elongation index of actively transcribed mRNAs (n = 7213) from BRD2 degron mESCs upon indicated treatments compared to DMSO control. **i**, PRO-seq analysis depicting log_2_FC of RNA Pol II coverage upon combined depletion of BRD2 and BRD4 in comparison to BRD4 depletion alone over all expressed mRNAs (n = 2). **j**, Gene Ontology enrichment analysis of BRD2 interactors defined in Fig. 4a. **k**,**l**, Histogram (left) and empirical cumulative distribution function (ECDF) plot (right) showing the distribution of Pol II cluster lifetime before and after rapid BRD2 (**k**) or BRD4 (**l**) depletion. The number of clusters analyzed is indicated in the panel. P-values were calculated using a two-sided Wilcoxon rank test. **m**, Representative super-resolved live-cell images of endogenously tagged TAF1-Halo in BRD2 degron mESCs upon acute BRD4 depletion. *n* = *2* independent experiments. **n**, Box plot depicting the changes in the number of TAF1 clusters upon BRD4 depletion as percentage of untreated condition (left). P-values were calculated using a two-sided t-test. Histogram and empirical cumulative distribution function (ECDF) plot showing the distribution of TAF1 cluster lifetime upon BRD4 loss (right). P-values were calculated using a two-sided Wilcoxon rank test. The box plots in **d**–**h**,**n** represent the IQR, spanning from the first quartile to the third quartile, with the median shown as a line inside the box. The whiskers extend to the minimum and maximum values in the datasets in **n** while they extend to the most extreme data points within 1.5× IQR in **d**–**h**. Source data

We estimated transcription start regions (TSRs) from PRO-seq data using tsrFinder^30^ and analyzed 5′ reads over these regions to estimate relative transcription initiation (Supplementary Note 3). Acute BRD2 depletion or its codepletion with BRD4 reduced 5′ PRO-seq reads over the TSRs compared to DMSO control or BRD4 depletion, respectively, with a general trend toward reduced transcription from TSRs (Fig. 3h). BRD2 depletion significantly reduced transcription from 3,435 of 9,768 analyzed TSRs, whereas BRD4 depletion had minimal impact (Fig. 3i and Extended Data Fig. 5a). In agreement with this, PRO-seq 3′ reads most prominently decreased in the prepause window upon BRD2 loss alone or with BRD4 codepletion. In contrast, BRD4 depletion alone increased PRO-seq reads over the pausing sites, possibly due to increased dwelling time of Pol II at the promoters (Extended Data Fig. 5b–d).

Reduced transcription initiation correlated with the reduction in RNA synthesis rates and GB Pol II occupancy (Extended Data Fig. 5e,f). Despite lower promoter Pol II occupancy and TSR transcription, RNA synthesis and GB Pol II occupancy were less severely affected upon BRD2 depletion. Because promoter-proximal Pol II pausing is the rate-limiting step for transcription, we hypothesized that transcriptional units, which have higher dependency on BRD2 and transcription initiation, may have lower Pol II pausing. Accordingly, genes with lower pausing indices were more severely affected in RNA synthesis upon BRD2 loss (Extended Data Fig. 5g).

Consistent with the well-established role of BRD4 in promoting productive transcription elongation, BRD4 depletion reduced elongation index while BRD2 loss had only a limited impact^8,10,26^. Codepletion of BRD2 and BRD4 further reduced elongation rates, supporting a functional collaboration of these two factors (Extended Data Fig. 5h).

We observed a similar trend of differential Pol II occupancy at the promoters and GBs of the differential genes from Fig. 2k upon codepletion of BRD2 and BRD4 in comparison to that of BRD4 depletion alone (Fig. 3j and Extended Data Fig. 5i). Our findings indicate that BRD2 depletion principally affects transcription initiation.

### BRD2 regulates the dynamics of pre-initiation complex and Pol II

**Fig. 4:**
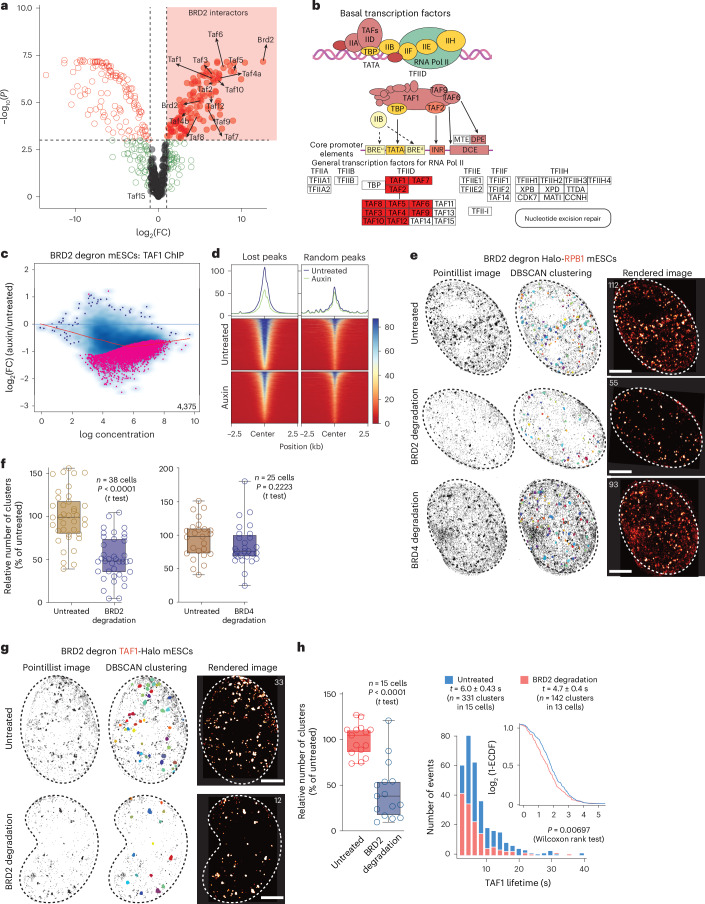
BRD2 regulates dynamic organization of basal transcription machinery. **a**, Volcano plot depicting significant BRD2 interactors (log_2_(FC) > 1 and FDR < 0.001) identified by mass spectrometry in comparison to the IgG negative control. *P* values were obtained from linear models fitted with limma, followed by Benjamini–Hochberg correction for multiple testing. **b**, Kyoto Encyclopedia of Genes and Genomes graph^48^ depicting the ‘basal transcription factors’ category with significant BRD2 interactors marked in red. **c**, MA plots of differential chromatin enrichment analysis for TAF1 upon acute BRD2 depletion (*n* = 2). Pink dots depict differentially enriched peaks with FDR < 0.05. **d**, Metagene plot and heatmap of ChIP–seq signal for TAF1 upon acute BRD2 depletion. The signal was plotted over the differential TAF1 ChIP–seq peaks obtained from **c**. **e**, Representative tcPALM super-resolved live-cell images of Halo-RPB1 in BRD2 degron mESCs with endogenous Halo-RPB1 upon BRD2 and BRD4 acute depletion (*n* = 3 independent experiments). Scale bars, 2 µm. **f**, Box plot depicting the changes in the number of RNA Pol II clusters upon BRD2 and BRD4 depletions as percentage of untreated condition. *P* values were calculated using a two-sided *t* test. **g**, Representative super-resolved live-cell images of endogenously tagged TAF1-Halo in BRD2 degron mESCs upon acute BRD2 depletion (*n* = 3 independent experiments). Scale bars, 2 µm. **h**, Box plot depicting the changes in the number of TAF1 clusters upon BRD2 depletion as percentage of untreated condition (left). *P* values were calculated using a two-sided *t* test. Histogram and ECDF plot showing the distribution of TAF1 cluster lifetime upon BRD2 loss (right). *P* values were calculated using a two-sided Wilcoxon rank test. The box plots in **f** and **h** represent the IQR, spanning from the first quartile to the third quartile, with the median shown as a line inside the box. The whiskers extend to the minimum and maximum values in the datasets. ECDF, empirical cumulative distribution function. DBSCAN, density-based spatial clustering of applications with noise. Source data

Mass spectrometry after immunoprecipitation of endogenous BRD2 identified components of basal transcription machinery as the strongest interactors of BRD2. Multiple TFIID subunits were enriched upon BRD2 pull-down in line with the role of BRD2 in transcription initiation (Fig. 4a,b, Extended Data Fig. 5j and Supplementary Table 1). Furthermore, BRD2 depletion led to a reduction of TAF1 chromatin enrichment (Fig. 4c,d).

Our results indicated that BRD2 depletion or loss of its chromatin binding rapidly alters Pol II enrichment at the promoters. We performed live-cell super-resolution microscopy to study the real-time responses of Pol II to rapid BRD2 depletion at the single-cell level. We engineered endogenous Halo-Rpb1 in BRD2 degron mESCs and analyzed subdiffraction Pol II clusters, which are known to correlate with transcription initiation^31,32^. Time-correlated photoactivation localization microscopy (tcPALM) identified Pol II clusters with median lifetimes of 6.5–7.5 s (Fig. 4e and Extended Data Fig. 5k,l)^31^. BRD2 depletion led to an average reduction of ~50% Pol II clusters, without severely affecting the half-life of the remaining clusters, suggesting that not all clusters depend on BRD2 (Fig. 4f and Extended Data Fig. 5k). BRD4 depletion did not change the number of Pol II clusters but modestly increased their lifetime, highlighting the roles of BRD2 and BRD4 in related, but different layers of transcription regulation (Fig. 4e,f and Extended Data Fig. 5l).

To measure single-cell dynamics of the pre-initiation complex, we endogenously tagged TFIID subunit TAF1 with a Halo tag. Similar to Pol II, Halo-TAF1 also formed short-lived dynamic clusters with an average half-life of ~6.0 s (Fig. 4g,h). BRD2 depletion diminished TAF1 clusters, while acute BRD4 depletion had no effect, providing further evidence for the role of BRD2 in the regulation of transcription initiation dynamics (Fig. 4g,h and Extended Data Fig. 5m,n).

### Differential clustering properties of BRD2 and BRD4

**Fig. 5:**
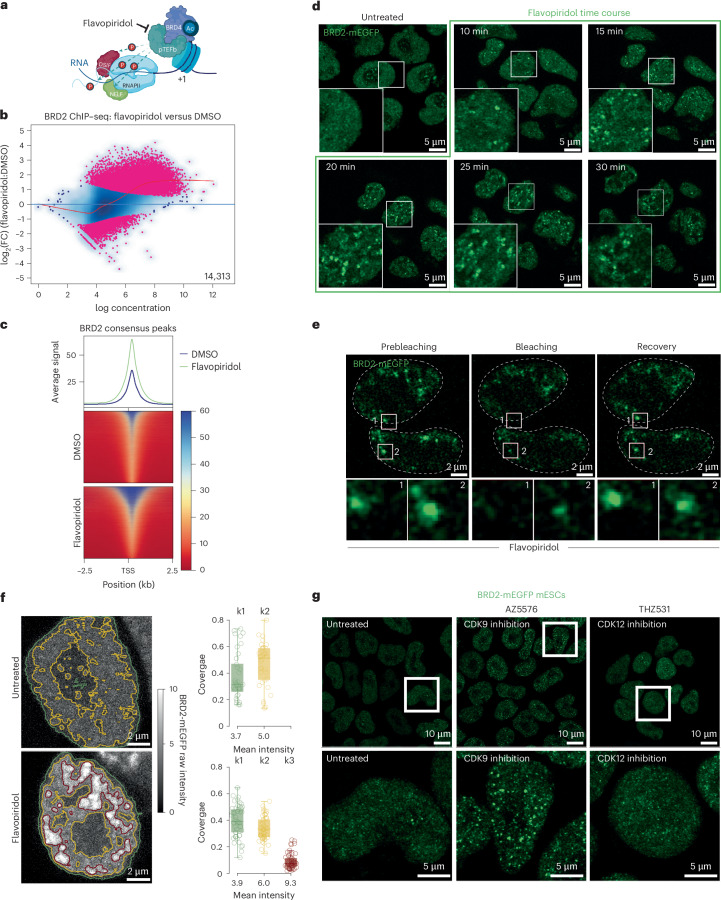
BRD2 reorganizes into dynamic foci upon inhibition of pause release. **a**, Schematic describing the experimental strategy to study the function of BRD2 in relation to the dynamics of paused RNA Pol II using flavopiridol-mediated inhibition of pTEFb catalytic activity. DSIF, DRB sensitivity inducing factor; NELF, negative elongation factor. Schematic in **a** created in BioRender; Erdogdu, U. https://biorender.com/r2eiulu (2026). **b**, MA plots of differential chromatin enrichment analysis for BRD2 upon flavopiridol treatment of BRD2 degron mESCs for 1 h (*n* = 2). Pink dots depict differentially enriched peaks with FDR < 0.05. **c**, Metagene plots and heatmap of ChIP–seq signal for BRD2 upon flavopiridol treatment. The signal was plotted over BRD2 consensus peaks. **d**, Time-lapse live-cell fluorescence microscopy images of BRD2-mEGFP signal in untreated and flavopiridol-treated (for the indicated time points) mESCs. **e**, Representative images depicting the fluorescence recovery of BRD2-mEGFP foci after photobleaching. **f**, *K*-means classification of pixel intensities from live-cell images of cells treated with flavopiridol for 30 min. A contour plot showing each of the clusters detected (left) and a box plot depicting nuclear coverage of each cluster detected (right) are shown. The central line in the box plots indicates the median while the lower and upper edges of the box correspond to the 25th and 75th percentiles, respectively. The whiskers extend to the most extreme data points within 1.5× IQR (*n* = 35 cells (untreated), *n* = 53 cells (flavopiridol)). **g**, Live-cell fluorescence microscopy images of BRD2-mEGFP upon treatment with AZ5576 and THZ531. Source data

**Extended Data Fig. 6:**
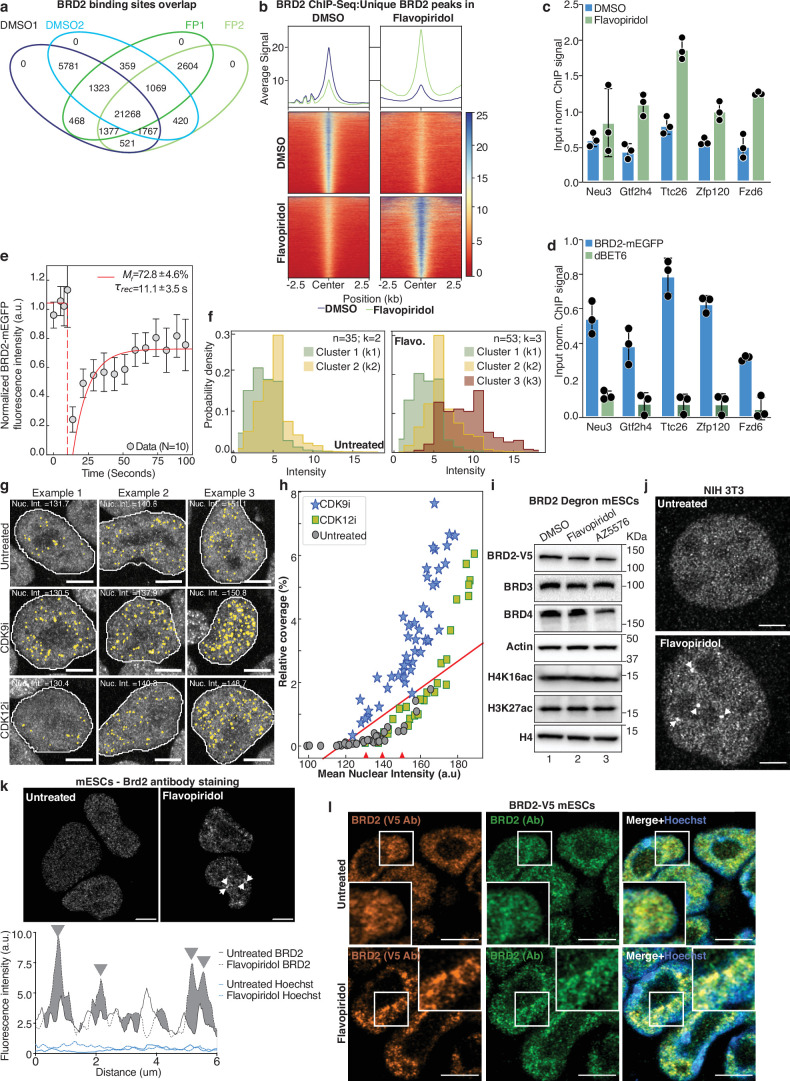
BRD2 accumulates on the chromatin and forms foci upon pause release inhibition. **a**, Venn diagram showing overlap of BRD2 binding sites between individual replicates of DMSO and flavopiridol-treated BRD2-V5 mESCs (n = 2). **b**, Metagene profiles and heatmaps depicting the spike-in-normalized ChIP–seq signal of BRD2 upon flavopiridol-treated BRD2–V5 mESCs. The signal was plotted over BRD2 peaks which were unique in DMSO control or in flavopiridol-treated cells. **c**, BRD2 ChIP–qPCR of selected BRD2 target genes in untreated and flavopiridol-treated Hepa 1-6 cells. Data indicate mean ± s.e.m., n = 3 biological replicates. The data were normalized to input. **d**, BRD2 ChIP–qPCR of selected BRD2 binding sites in untreated and dBET6-treated BRD2-mEGFP mESCs, confirming signal specificity. Data indicate mean ± s.e.m., n = 3 biological replicates. The data were normalized to input. **e**, The FRAP curve from live-cell imaging in Fig. 5e showing mean ± s.e. across 10 independent clusters for individual time points. **f**, Histogram of the contour plot in Fig. 5f, depicting probability densities of pixel intensities in each cluster detected. **g**, Selection of cells from untreated, CDK9 inhibited and CDK12 inhibited BRD2-mEGFP mESCs. Cellpose-derived nuclear masks are shown in white. Yellow contours mark the highest-intensity cluster, identified using K-means from sklearn.cluster. The average nuclear intensity (Nuc. Int.) is indicated for each case. Scale bar of images: 2 μm. **h**, Scatterplot showing coverage of the condensed state vs. mean nuclear intensity from **g**. Red arrowheads at the bottom correspond to the nuclear intensity values of the images shown. **i**, Immunoblot analysis of DMSO, flavopiridol and CDK9 inhibitor (AZ5576) treated BRD2 degron mESCs using antibodies against BET proteins and histone marks. Actin and total histone H4 served as loading controls. **j**, Immunofluorescence microscopy images of untreated and flavopiridol-treated NIH-3T3 mouse fibroblasts using an antibody against BRD2 (white). White arrows indicate the BRD2 foci. Scale bar of images: 5 μm. **k**, Immunofluorescence microscopy images (top) and line profile (bottom) of untreated and flavopiridol-treated mESCs using an antibody against BRD2 (white). White arrows in the image and gray arrows in the line profile indicate the BRD2 foci. Scale bar of images: 5 μm. **l**, Immunofluorescence microscopy images of untreated and flavopiridol-treated BRD2-V5 mESCs using an antibody against V5 (orange), BRD2 (green) and nuclear Hoechst staining (blue). Scale bar of images: 5 μm. Source data

Given the more prominent role of BRD2 in the control of Pol II progression in the absence of BRD4, we asked whether there might be a dynamic interplay between BRD2 and paused Pol II. To this end, we inhibited Pol II pause release using the pTEFb inhibitor flavopiridol and found a visible increase in BRD2 chromatin binding (Fig. 5a–c). Although most BRD2 ChIP–seq peaks were common between DMSO and flavopiridol treatments, there were 2,604 de novo BRD2 peaks upon flavopiridol treatment (Extended Data Fig. 6a,b). We also observed a similar increase in BRD2 chromatin binding in Hepa 1-6 cells upon flavopiridol treatment (Extended Data Fig. 6c).

To examine BRD2 dynamics at single-cell resolution, we endogenously tagged BRD2 at its C terminus with mEGFP (Extended Data Figs. 3a and 6d). Under steady-state conditions, BRD2 was relatively homogenous in the nucleus. However, flavopiridol treatment induced dynamic foci that underwent continuous dissolving and reformation (Fig. 5d and Supplementary Video 1). Fluorescence recovery after photobleaching experiments confirmed rapid recovery of BRD2-mEGFP foci within 30 s (Fig. 5e and Extended Data Fig. 6e). We could also quantify the emergence of the flavopiridol-induced BRD2 foci by performing *k*-means clustering analysis of the BRD2-mEGFP signal (Fig. 5f and Extended Data Fig. 6f), corroborating the increased BRD2 chromatin binding after flavopiridol treatment (Fig. 5b–d). Cdk9 inhibition with AZ5576, but not Cdk12/Cdk13 inhibition with THZ531, also induced BRD2 foci (Fig. 5g and Extended Data Fig. 6g–i). We could also observe flavopiridol-induced BRD2 foci in untagged mESCs and NIH-3T3 cells, albeit to a different extent (Extended Data Fig. 6j–l and Supplementary Note 4).

### H4ac- and intrinsically disordered region (IDR)-mediated BRD2 clustering regulates BRD2 chromatin enrichment

**Fig. 6:**
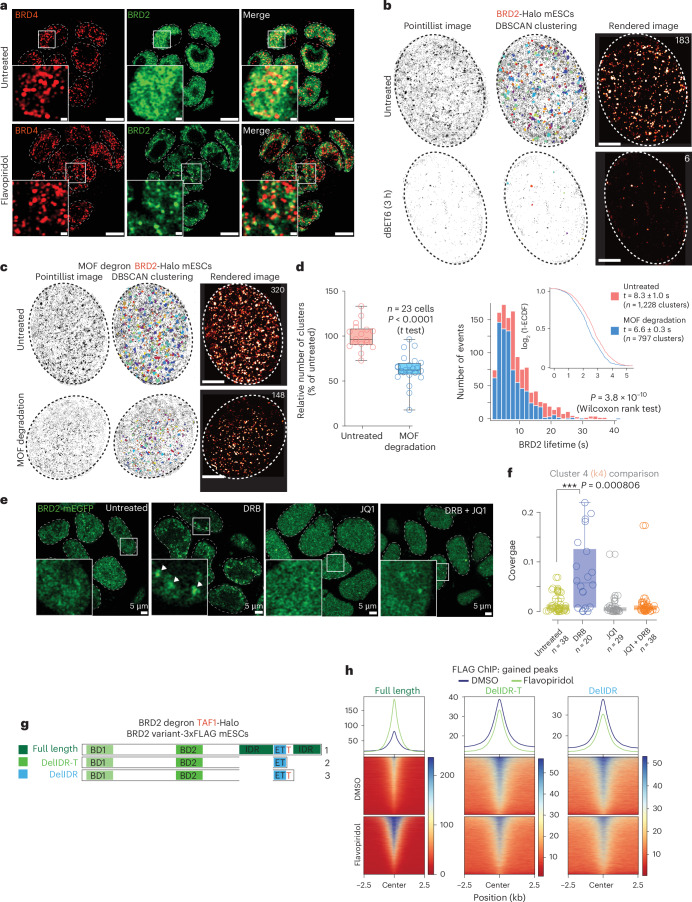
Histone acetylation modulates IDR-driven clustering dynamics of BRD2. **a**, Immunofluorescence microscopy images of untreated and 1-h flavopiridol-treated BRD2 degron mESCs stained using antibodies against BRD4 (red) and V5 (green) for BRD2–mAID–V5. Scale bars, 5 µm; insets, 500 nm. **b**, Example of a super-resolved live-cell image of BRD2-Halo in mESCs. Two-dimensional super-resolution reconstruction reveals the presence of BRD2 clusters that disappear upon dBET6-mediated degradation of BRD2. The numbers on top right of the rendered images indicate the number of BRD2 clusters in the representative cells detected by DBSCAN. Scale bars, 2 µm. **c**, Example of super-resolved live-cell images of BRD2 in BRD2-Halo MOF degron mESCs before and after auxin treatment. Scale bars, 2 µm. **d**, Left, box plot depicting the changes in the number of BRD2 clusters upon MOF depletion as a percentage of the untreated condition (*n* = 23 cells). The box plot represents the IQR, spanning from the first quartile to the third quartile, with the median shown as a line inside the box. The whiskers extend to the minimum and maximum values in the datasets. *P* values were calculated using a two-sided *t* test. Right, histogram and ECDF plot showing the distribution of BRD2 cluster lifetime before and after rapid MOF depletion. *P* values were calculated using a two-sided Wilcoxon rank test. **e**, Live-cell fluorescence microscopy images of BRD2-mEGFP mESCs upon single or combined treatments with DRB and JQ1 for 1 h. **f**, Box plot depicting coverage of high-intensity cluster 4 (k4) per cell in BRD2-mEGFP mESCs that were either untreated DRB or treated with JQ1, DRB or JQ1 + DRB. The central line in the box plot indicates the median, while the lower and upper edges of the box correspond to the 25th and 75th percentiles, respectively. The whiskers extend to the most extreme data points within 1.5× IQR. *P* values were calculated using a two-sided Welch’s two-sample *t* test (*n* = 38 cells (untreated), *n* = 20 cells (DRB), *n* = 29 cells (JQ1), *n* = 38 cells (DRB + JQ1)). **g**, Schematic depicting the domain organization of BRD2 variants. **h**, Metagene plots and heatmaps depicting the chromatin enrichment of BRD2 variants expressed in BRD2 degron mESCs upon doxycycline induction and overnight depletion of endogenous BRD2. Cells were treated with flavopiridol for 1 h. Signal was plotted over BRD2 peaks from Fig. 5b (*n* = 2). Source data

Given that BRD4 forms stable condensates in mESCs, we asked whether BRD2 foci colocalize with BRD4 condensates^33^. Immunofluorescence upon flavopiridol treatment showed minimal overlap between BRD2 foci and BRD4 clusters, implying distinct nuclear dynamics of these two proteins (Fig. 6a).

**Extended Data Fig. 7:**
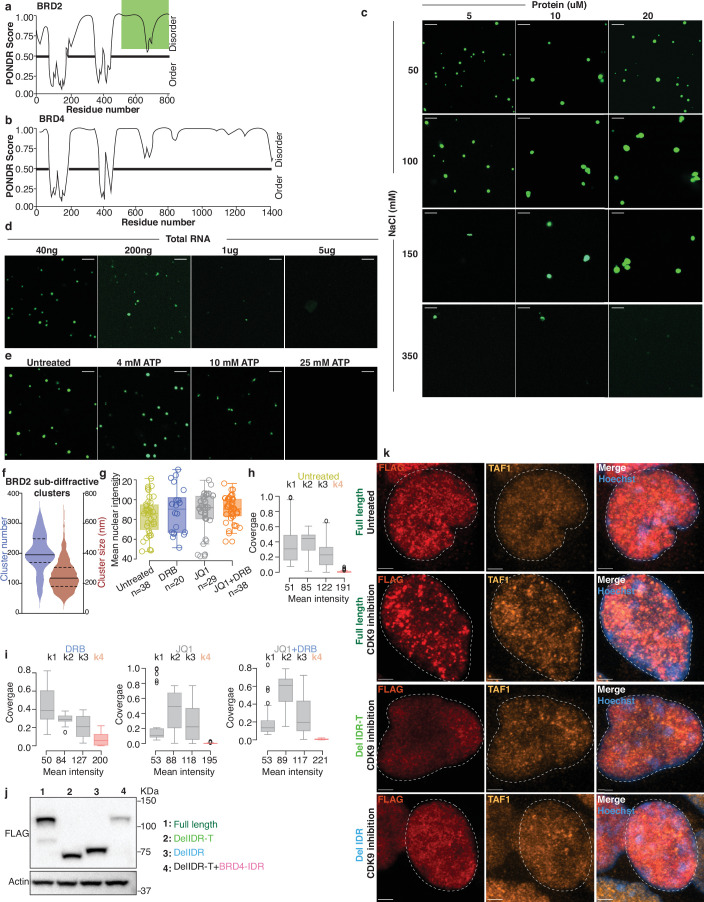
In vitro characterization of BRD2 IDR and its role in BRD2 clustering. **a**,**b**, Graphs plotting the intrinsic disorder score for BRD2 (**a**) and BRD4 (**b**). PONDR (Predictor of Natural Disordered Regions) VSL2 scores, that is, neural network predictions of short and long disordered regions, are shown on the y axis and amino acid positions are shown on the x axis. The green box in **a** designates the IDR investigated in the lower panels. **c**, Fluorescence microscopy images of the mEGFP-tagged IDR of BRD2 at indicated protein concentrations in buffers with variable NaCl concentrations. Scale bar of images: 5 µm. **d**,**e**, Fluorescence microscopy images of the purified mEGFP-tagged IDR of BRD2 in buffers with variable total RNA (**d**) or ATP concentrations (**e**). Scale bar of images = 5 µm. **f**, Violin plots depicting the BRD2 cluster number (blue) and the cluster size (red) obtained from live-cell super-resolution microscopy of BRD2-Halo mESCs. **g**, Box plot showing distribution of nuclear intensities in untreated, DRB or JQ1 treated BRD2-mEGFP mESCs (Fig. 6e). The number of replicates analyzed is indicated below each box. **h**,**i**, Box plot for clustering of the pixels from the experiment in Fig. 6e into 4 clusters, showing high-intensity clusters present more prominently in the DRB perturbation case. This is shown as the coverage (fraction of nuclear area occupied by the high-intensity regions). The box plots in **g**–**i** represent the IQR, spanning from the first quartile to the third quartile, with the median shown as a line inside the box. The whiskers extend to the most extreme data points within 1.5× IQR. **j**, Western blot showing the expression of 3×FLAG-tagged BRD2 variants in BRD2 degron mESCs for the experiments in **k** and Figs. 6g,h and 7a,b. Actin serves as a loading control. **k**, Immunofluorescence microscopy images of untreated and flavopiridol-treated BRD2 degron mESCs stably expressing BRD2 variants described in Fig. 6g, using an antibody against FLAG (red), TAF1 (orange) and nuclear Hoechst (blue). Scale bar of images: 5 μm. Source data

The IDR of BRD4 is larger than that of BRD2, despite their similar domain organization (Extended Data Fig. 7a,b). Nevertheless, the BRD2 IDR also phase-separated in vitro, forming condensates sensitive to both ATP and RNA (Extended Data Fig. 7c–e). We hypothesized that BRD2 forms short-lived clusters not detectable by confocal microscopy. To test this, we endogenously tagged BRD2 with a Halo tag and observed its dynamics using live-cell super-resolution microscopy. tcPALM revealed transient BRD2 clusters, with an average size of ~250 nm and ~200 clusters per cell, which disappeared upon dBET6 treatment (Fig. 6b and Extended Data Fig. 7f).

Because MOF depletion prominently affects BRD2 chromatin binding (Fig. 1e), we asked whether BRD2 transient clusters also depend on H4ac. We tagged the endogenous *Brd2* locus with Halo in MOF degron mESCs and performed live-cell super-resolution microscopy. MOF depletion reduced BRD2 clusters by ~50% and destabilized them, as evident from their reduced average half-life (Fig. 6c,d). Similarly, JQ1 treatment diminished the frequency of BRD2 foci induced by pause-release inhibition (DRB + JQ1 versus DRB; Fig. 6e,f and Extended Data Fig. 7g–i), implying that acetylated chromatin is required for the spatial organization of BRD2.

To further investigate the role of the BRD2 IDR in its chromatin dynamics, we generated BRD2 degron mESCs expressing an IDR-less BRD2 variant (DelIDR-T) under a doxycycline-inducible promoter (Fig. 6g and Extended Data Fig. 7j). Deletion of the BRD2 IDR abolished flavopiridol-induced foci (Extended Data Fig. 7k). A ~10-amino-acid-long coiled-coil domain embedded within the BRD2 IDR (named as ‘T’ domain) was previously predicted^34^. Therefore, we created a variant lacking the IDR, while retaining the T domain (named as the DelIDR variant) as well. This variant also failed to form foci upon flavopiridol treatment (Fig. 6g and Extended Data Fig. 7j,k). Only full-length BRD2, but not the IDR or IDR-T deletion variants, showed increased chromatin binding upon pause-release inhibition by flavopiridol (Fig. 6h). These findings suggest that local BRD2 chromatin enrichment is primarily driven by its IDR-dependent clustering and H4ac-mediated recruitment.

### IDR-dependent and H4ac-dependent local enrichment of BRD2 on chromatin is required for its function

**Fig. 7:**
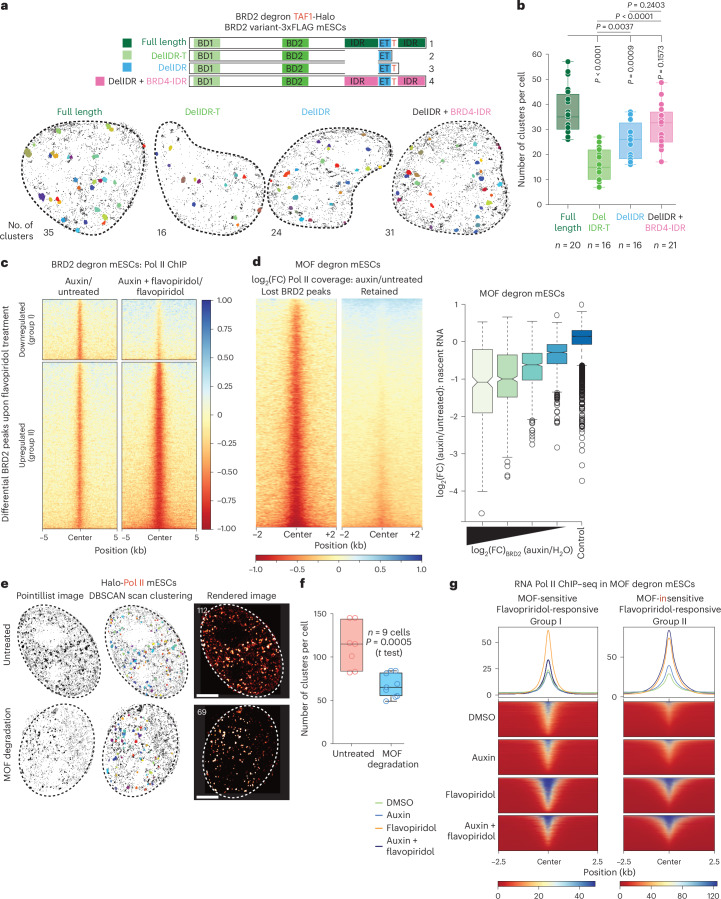
Functional role of BRD2 clustering in MOF-driven transcriptional dynamics. **a**, BRD2 variant scheme (top) and representative tcPALM super-resolved TAF1 clusters identified by DBSCAN clustering in each variant (*n* = 3 independent experiments). **b**, Box plot depicting the changes in the number of TAF1 clusters in BRD2 degron mESCs expressing BRD2 variants in the absence of endogenous BRD2 (*n* = 3 independent experiments). *P* values were calculated using Brown–Forsythe and Welch ANOVA, followed by Dunnett’s T3 multiple comparisons test with separate variances for each comparison. **c**, ChIP–seq analysis depicting the log_2_(FC) of RNA Pol II coverage upon rapid BRD2 depletion with (right) and without (left) flavopiridol treatment. The signal was plotted over the differential BRD2 ChIP–seq peaks obtained from Fig. 5b. **d**, ChIP–seq analysis depicting the log_2_(FC) of RNA Pol II coverage upon 3 h of auxin treatment of MOF degron mESCs for 3 h. The signal was plotted over the BRD2 ChIP–seq peaks that were lost or retained upon MOF depletion (left) and box plot depicting the log_2_(FC) of nascent RNA levels in relation to differential BRD2 enrichment over their promoters upon rapid MOF depletion. All expressed genes that exhibited differential BRD2 binding at their promoters after MOF depletion (*n* = 4,676) were sorted into four quartiles of equal size based on the log_2_(FC) of BRD2 occupancy upon MOF depletion. The expressed genes without loss of BRD2 enrichment at their promoters (*n* = 2,072) served as a control (dark blue; right). **e**, Representative tcPALM super-resolved live-cell images of Halo-RPB1 in MOF degron mESCs with endogenous Halo-RPB1 upon acute MOF depletion (*n* = 3 independent experiments). Scale bars, 2 µm. **f**, Box plot depicting the changes in the number of RNA Pol II clusters upon MOF depletion as percentage of untreated condition. *P* values were calculated using a two-sided *t* test. **g**, Metagene plots and heatmap depicting RNA Pol II occupancy in MOF degron mESCs upon flavopiridol treatment and acute MOF depletion. The signal was plotted over the promoters of the gene groups defined in Extended Data Fig. 8f. The box plots in **b**, **d** and **f** represent the IQR, spanning from the first quartile to the third quartile, with the median shown as a line inside the box. The whiskers extend to the minimum and maximum values in the datasets in **b** and **f**, while they extend to the most extreme data points within 1.5× IQR in **d**. ANOVA, analysis of variance. Source data

**Extended Data Fig. 8:**
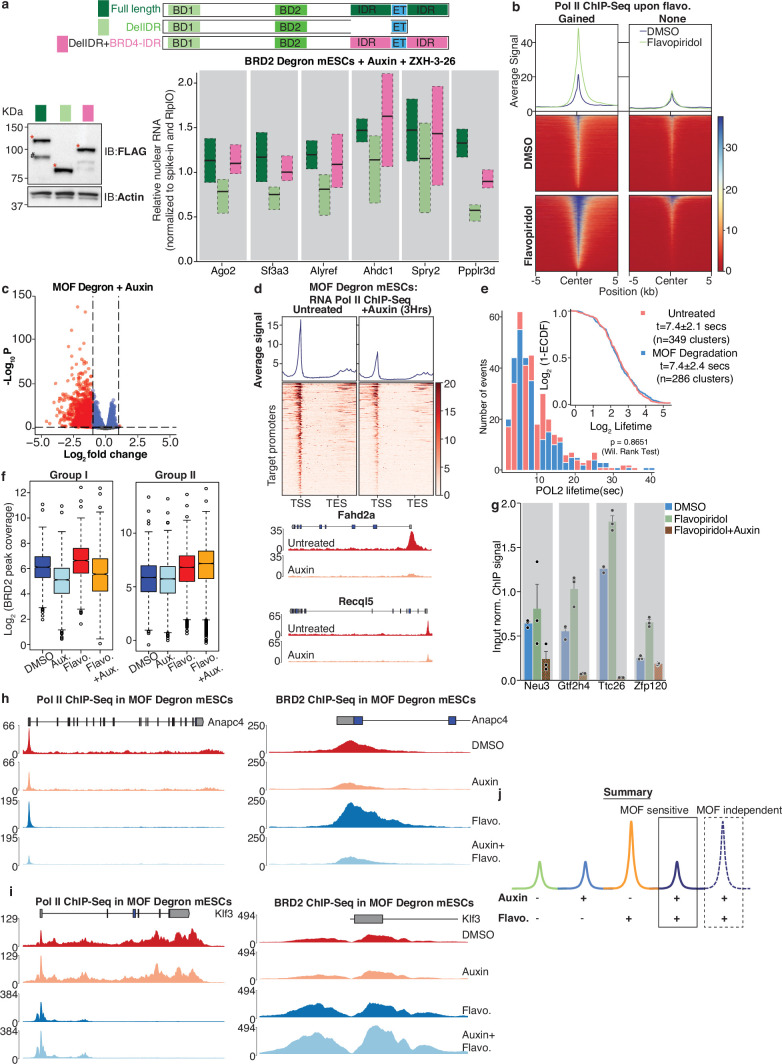
The role of BRD2 clustering driven by H4 acetylation in transcription regulation. **a**, Schematic depicting the domain organization of BRD2 variants (top). All the mutants carry the two BRD2 bromodomains and the BRD2 ET domain. They only differ in their IDRs. For replacement of the BRD2 IDR with an exogenous IDR, a segment of the BRD4 IDR with a comparable size to the original BRD2 IDR was used. Western blot (bottom left) showing the expression of 3×FLAG-tagged BRD2 variants in cells. Red asterisks denote the specific bands. Actin serves as a loading control. Box plots (bottom right) depicting the changes in nuclear RNA levels of the mentioned transcripts upon expression of BRD2 variants in the absence of endogenous BRD2 and BRD4 (n = 3 biological replicates). The central line in the box plot represents the mean value, while its lower and upper edges correspond to the minimum and maximum values. **b**, Heatmap and metagene plots depicting RNA Pol II enrichment upon flavopiridol treatment. The signal was plotted over the promoters of the genes with increased chromatin binding of BRD2 upon pause release inhibition in comparison to those with BRD2 not responding to flavopiridol treatment from (Fig. 5b) (n = 2). **c**, Volcano plot showing differentially expressed genes identified by TT-seq (red) upon 3-h auxin treatment of MOF degron mESCs. The dashed lines mark the log_2_FC-cutoff of 1 (vertical lines) and adjusted-p value cutoff of 0.05 (horizontal lines). Statistical significance was determined using a Wald test implemented in DESeq2, followed by correction for multiple comparisons using the Benjamini–Hochberg method. **d**, Metagene plot, heatmap (top) and example genome snapshots (bottom) of spike-in-normalized RNA Pol II ChIP–seq signal from MOF degron mESCs upon auxin treatment for 3 h. The signal was plotted over the differentially expressed genes obtained from **c**. **e**, Histogram and empirical cumulative distribution function (ECDF) plot showing the distribution of Pol II cluster lifetime upon MOF degradation. P-value was calculated using a two-sided Wilcoxon rank test. **f**, Box plots depicting log_2_ (BRD2 peak coverage) upon treatment of MOF degron mESCs with auxin and flavopiridol. BRD2 peaks were sorted into two groups based on their response to MOF depletion (group I: 2747 peaks, group II: 9070 peaks). The box plots represent the IQR, spanning from the first quartile to the third quartile, with the median shown as a line inside the box. The whiskers extend to the most extreme data points within 1.5× IQR. **g**, BRD2 ChIP–qPCR for selected genes in MOF degron mESCs upon auxin and flavopiridol treatments (n = 3 biological replicates). Data indicate mean ± s.e.m. and were normalized to input. **h**,**i**, Genome snapshots depicting the changes in RNA Pol II chromatin occupancy on example genes (left) and BRD2 chromatin binding at their promoters (right) from group I (**h**) and group II (**i**). **j**, Schematic summarizing the changes in RNA Pol II occupancy upon flavopiridol and auxin treatments. Source data

To assess the functional relevance of the BRD2 IDR, we performed tcPALM with TAF1-Halo BRD2 degron mESCs complemented with BRD2 variants. The IDR and IDR-T deletion variants failed to rescue TAF1 clusters when compared to BRD2-depleted cells complemented with full-length BRD2 (Fig. 7a,b). To determine whether the BRD2 IDR is functionally replaceable, we generated a chimeric BRD2 variant containing a segment of the BRD4 IDR that is similar in size to the BRD2 IDR (named DelIDR + BRD4 IDR). This variant partially rescued TAF1 cluster formation, despite its lower expression levels (Fig. 7a,b and Extended Data Fig. 7j). Furthermore, only full-length BRD2 and the chimeric variant could partially rescue the expression of selected target genes upon codepletion of BRD2 and BRD4 (Extended Data Fig. 8a).

To investigate the regulatory function of BRD2 in transcription in relation to its increased chromatin association upon flavopiridol treatment, we compared differential Pol II coverage around the BRD2 peaks that exhibited either reduced (group I peaks) or increased (group II peaks) enrichment upon pause-release inhibition (Figs. 5b and 7c). As observed previously (Fig. 3a), BRD2 depletion caused a mild reduction in Pol II occupancy under steady-state conditions in both groups (Fig. 7c, left). However, upon pause-release inhibition, BRD2 loss led to a stronger reduction in Pol II occupancy around group II peaks (Fig. 7c, bottom right) compared to group I peaks (Fig. 7c, top right). These findings imply a role of BRD2 in maintaining the chromatin occupancy of paused Pol II. Consistent with this, promoters with BRD2 peaks gaining enrichment upon flavopiridol treatment exhibited overall higher Pol II occupancy (Extended Data Fig. 8b).

Next, we investigated the transcriptional impact of MOF depletion in relation to BRD2 chromatin binding. Acute MOF depletion led to significant transcriptional downregulation of 1,086 genes, accompanied by reduced promoter Pol II occupancy (Extended Data Fig. 8c,d). Consistent with earlier observations (Fig. 3a–c), Pol II coverage was more severely reduced around MOF-sensitive BRD2 peaks (Fig. 7d, left), correlating with a more severe reduction in RNA synthesis (Fig. 7d, right). Similar to BRD2 depletion, MOF loss also reduced the number of Pol II clusters (Fig. 7e,f and Extended Data Fig. 8e).

We also investigated whether H4ac is a prerequisite for the increased chromatin enrichment of BRD2 upon pause-release inhibition. We identified two major groups of flavopiridol-enriched BRD2 peaks with differing sensitivity to MOF depletion (Extended Data Fig. 8f,g)—while group I peaks were sensitive to MOF depletion under both control condition and flavopiridol treatment, group II peaks were not affected. Pol II chromatin occupancy showed a higher MOF dependency at the promoters of group I genes upon flavopiridol treatment. In contrast, MOF depletion led to slightly higher Pol II occupancy at the promoters of group II genes (Fig. 7g and Extended Data Fig. 8h–j). These findings further highlight the functional relationship between H4ac and BRD2 in controlling Pol II dynamics at promoters.

## Discussion

**Extended Data Fig. 9:**
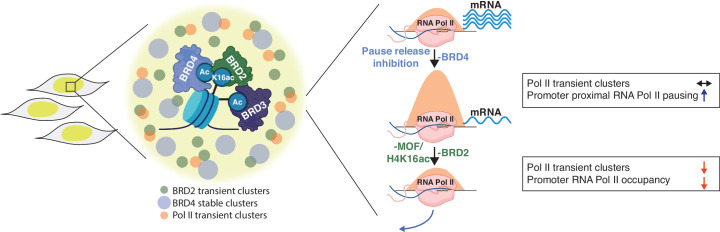
A schematic working model depicting the role of BRD2 in the control of RNA Pol II dynamics. In the nucleus, the transcription machinery and the transcriptional co-activators are organized into distinct clusters with discrete spatiotemporal dynamics. While BRD2 and RNA Pol II form transient clusters, BRD4 forms stable condensates. Upon inhibition of pause release through either depletion of BRD4 or small molecule treatment such as flavopiridol, the dynamics of transcriptional machinery change: BRD2 organizes itself into dynamic foci and exhibits increased chromatin binding through an IDR-mediated condensation mechanism. RNA Pol II transient clusters persist and loaded RNA Pol II accumulates at the promoter proximal site in the paused state. In this situation, loss of BRD2 diminishes promoter proximal enrichment of RNA Pol II by reducing transcription initiation. Concomitantly, BRD2 depletion also disrupts Pol II transient clusters. H4 acetylation mediated by MOF is an important regulator of BRD2 recruitment to the chromatin and its acute depletion recapitulates the consequences of BRD2 depletion in relation to RNA Pol II clustering and promoter RNA Pol II occupancy. Given the anticorrelation between RNA levels and BRD2 IDR in vitro condensation, we propose that BRD2 acts as a sensor of prolonged pausing and counteracts possible loss of paused RNA Pol II occupancy at the promoters through premature termination by promoting transcription initiation. Schematics for BET proteins, Pol II and RNA created in BioRender; Erdogdu, U. https://biorender.com/2kg2baf (2026).

In this study, we dissected the function of BRD2 as a reader of histone H4ac and its hierarchical interplay with other BET proteins. Integrating genomics and super-resolution microscopy, we unraveled the role of BRD2 clustering in maintaining chromatin occupancy of Pol II at promoters, which becomes more crucial in the absence of BRD4 or upon pause-release inhibition (Extended Data Fig. 9).

Codepletion experiments reveal functional segregation of BET proteins in transcription regulation—while BRD4 acts as an essential regulator of transcription elongation by promoting the release of Pol II from promoter-proximal pausing, BRD2 promotes transcription initiation in line with previous reports^10,11,33^. Our BRD2 interactome was strongly enriched for components of basal transcription machinery, particularly TFIID, consistent with previous proteomics studies^11,35^. BRD2 loss also reduces TAF1 chromatin occupancy and disrupts TAF1 transient clusters, impairing transcription initiation. Notably, acute TAF1 depletion was previously shown to trigger a muted decrease in transcription elongation despite the strong reduction in transcription initiation, suggesting that the transcription process can partially tolerate reduced transcription initiation, possibly by promoting pause release^30^. In this respect, acute depletion of BRD4 reveals the functional relevance of BRD2 in nascent transcription. Pol II chromatin occupancy upon BRD4 loss or pause-release inhibition largely depends on BRD2-mediated control of transcription initiation. Paused Pol II has also been suggested to have a fast turnover and very often undergo promoter-proximal termination^36–40^. Accordingly, before proceeding into productive elongation, Pol II goes through multiple cycles of initiation, pausing, termination and re-initiation. Productive transcription depends on both the number of Pol II molecules initiating and the fraction successfully released from the pause site. Even when pause release is strongly limiting and premature termination occurs at high rates, the absolute number of Pol II entering the pause region still determines how many molecules can potentially elongate. Thus, reducing initiation by BRD2 depletion further diminishes transcription because fewer Pol II are available to attempt pause release, likely explaining why combined BRD2 and BRD4 loss results in a more severe defect than either alone.

Our findings indicate that H4ac is not only essential for BRD2 chromatin recruitment but also dictates its spatial organization. BRD2 forms transient clusters under steady-state conditions and dynamic foci upon pause-release inhibition, which are sensitive to MOF depletion or JQ1 treatment. In line with this, it has been proposed that acetylated chromatin provides a multivalent platform for the clustering of BD proteins by reducing the concentration required for condensation^41–43^.

By linking transcriptional changes to BRD2 clustering and histone acetylation, we demonstrated the functional relevance of BRD2 clustering in transcription regulation. Consistent with prior work implicating the BRD2 C terminus in chromatin binding, deletion of the BRD2 IDR disrupted foci formation and abolished the increased chromatin binding observed upon pause-release inhibition^44^. The IDR-deleted variant also failed to rescue transient TAF1 clusters, supporting a functional role for BRD2 clustering in transcription dynamics. Furthermore, disruption of BRD2 chromatin binding through MOF depletion or loss of BRD2 led to more pronounced reductions in paused Pol II occupancy at promoters upon flavopiridol treatment.

Mechanistically, our data suggest that there is a dynamic interplay across promoter-proximal Pol II, BRD2 and BRD4. In the presence of BRD4 and very likely other transcriptional co-activators promoting rapid pause release, the function of BRD2 in promoting chromatin association of paused Pol II becomes less pronounced as a rapid transition into productive elongation takes place. By depleting BRD4 or inhibiting pause release using flavopiridol, we were able to take snapshots of the dynamic relationship between Pol II and BRD2. Similar to the increase in BRD2 chromatin binding upon pause-release inhibition, acute BRD4 depletion also leads to increased chromatin binding of BRD2 (ref. ^23^). In this respect, it is likely that nascent RNA counteracts the formation of stable BRD2 condensates as it has been previously proposed for BRD4 condensates^45^. In line with this, it has been shown that nascent RNA antagonizes the interaction of certain regulatory proteins with nucleosomes, including BRD2 (Supplementary Note 5)^46^.

Although BRD2 functionally interacts with TFIID in regulating promoter Pol II dynamics, whether this interaction is direct remains to be determined. To assess the functional contribution of BRD2 condensation, we generated BRD2 variants lacking or replacing its IDR. Whereas the IDR-deletion mutants were expressed normally, replacement with the BRD4 IDR resulted in reduced expression and only partial functional rescue, indicating that BRD2 and BRD4 condensation properties are not fully interchangeable. Future refinement of IDR designs will be required to resolve this aspect. Furthermore, our analysis focused on promoter-proximal functions of BRD2; however, BRD2 has also been implicated in enhancer regulation, suggesting broader context-dependent roles beyond those examined here^10,47^.

Collectively, our study provides an important example for the functional role of histone acetylation-dependent transcriptional co-activator condensation in transcription dynamics. Our data support a model by which the differential condensation properties of BRD2 and BRD4 give rise to distinct modules that support transcription initiation and elongation, respectively. The synergistic roles of BRD2 and BRD4 in transcription control also imply a crosstalk on the level of their condensation dynamics. Given the prominence of BET inhibitors in preclinical studies, our work provides a new mechanistic avenue to consider further developing their therapeutic efficacy.

## Methods

### Cell culture

WT26 male mESCs were a kind gift of Thomas Jenuwein. ESCs were cultured at 37 °C and 5% CO_2_ on 0.1% gelatin-coated (Merck, G1393) dishes and were maintained in DMEM (Gibco, 37966-021) supplemented with 15% heat-inactivated ESC-specific fetal bovine serum (PANSera ES, P30-2602), 100 U ml^−1^ penicillin and 100 U ml^−1^ streptomycin (Gibco, 15140-122), 1 mM glutamine (GlutaMax; Gibco, 35050061), 1 mM sodium pyruvate (Gibco, 11360-70), nonessential amino acids (Gibco, 11140050), ß-mercaptoethanol (Gibco, 31350-010) and in-house purified leukemia inhibitory factor. ESCs were frozen for storage in 90% fetal calf serum and 10% DMSO.

NIH-3T3 (gift from R. Kemler) and Hepa 1-6 cells (obtained from BIOSS Toolbox) were cultured at 37 °C and 5% CO_2_ and maintained in DMEM (Gibco, 37966-021) supplemented with 10% fetal calf serum (Anprotec, AC-SM-0143 for NIH-3T3 and AC-SM-0190 for Hepa 1-6), 100 U ml^−1^ penicillin and 100 U ml^−1^ streptomycin (Gibco, 15140-122).

All the cell lines were regularly tested negative for mycoplasma contamination.

### Generation of Tir1-expressing stable cell line

For stable expression of Tir1, the reporter TALEN system was used as previously described^49^. Briefly, a genome cassette containing a CAG promoter, cDNA sequence of *Tir1* (subcloned from a plasmid kindly provided by Zuber laboratory) along with 3×Myc-HA tag and a BGH-poly(A) signal, was cloned into a targeting vector (a kind gift of Bühler laboratory) that contains the homology arms for insertion into *Rosa26* locus. This repair template along with pRR-Puro reporter and heterodimeric ELD/KKR *FokI* domain plasmids targeting *Rosa26* locus were cotransfected into wild-type (WT) mouse ES cells using Lipofectamine 2000 (Invitrogen, 11668027) according to the manufacturer’s instructions. One day after transfection, ESCs were selected using 2 μg ml^−1^ puromycin (Gibco, A11138-03) for 36 h. Positive clones were picked using pipette tips and transferred into 96-well plates for further screening of Tir1 protein level.

### CRISPR–Cas9 genome editing

To endogenously tag proteins of interest with tags at their N or C termini, CRISPR–Cas9 genome editing was used. Guide RNAs were designed using CRISPR web toolbox CHOPCHOP^50^ (Supplementary Table 2). One microliter of each of 100 μM forward and reverse oligonucleotides containing the guide RNA sequences with the appropriate extensions for cloning were mixed with 1 μl of T4 PNK kinase (NEB, M0201), 1 μl of 10 mM ATP and 1 μl of 10× T4 DNA ligase buffer in total volume of 10 μl. This reaction was first incubated at 37 °C for 30 min, then at 95 °C for 5 min and finally cooled down to room temperature with a ramping rate of 0.1 °C s^−1^. The annealed oligos were diluted 1:200 in ddH_2_O and 2 μl of this dilution was used for ligation into 100 ng of PX458 (Addgene, 48138) or PX459 (Addgene, 62988) backbones that have been digested with *BbsI* (NEB, r3539) and gel purified, using T4 DNA ligase (NEB, M0202).

To generate homology-directed repair (HDR) templates, 5′ homology and 3′ homology arms were designed adjacent to the cut site of Cas9 with a minimum length of 750 bp. The homology arms, PCR-amplified from mouse genomic DNA, and the inserts were assembled together into the pJET1.2 backbone (Thermo Fisher Scientific, K1231) using Gibson Assembly (NEB; Supplementary Table 2). HDR template and sgRNA for N-terminal endogenous tagging of RPB1 with Halo were a kind gift of the Cissé laboratory^51^.

For genome editing, 3 μg of HDR template and 1 μg of sgRNA-containing Cas9 backbone were cotransfected into 1 × 10^6^ mESCs (seeded in each well of a 6-well plate) using Lipofectamine 2000 (Invitrogen, 11668027) according to the manufacturer’s protocol. Six hours after the transfection, the culture medium was exchanged and the mESCs were allowed to recover for at least another 6 h under standard growth conditions. Then the mESCs were replated on a 10-cm plate and selected with appropriate antibiotics for 4–7 days. The positive clones were picked using pipette tips and transferred into 96-well plates for PCR screening and further growth. For proteins that were endogenously tagged with a fluorescence marker, the above-described strategy was used with the difference that single mESCs positive for the fluorescence tag were FACS sorted into 96-well plates.

### Small-molecule treatments

To induce rapid degradation in the degron cells, they were treated with 500 μM of auxin (3-indole acetic acid; Sigma) in ddH_2_O. We have used the following inhibitor concentrations for the rest of the compounds: flavopiridol (10 μM in DMSO; Sigma), ZXH-3-26 (100 nM in DMSO; Tocris Bioscience), dBET6 (100 nM; Tocris Bioscience), DRB (100 μM in DMSO; Sigma), JQ1 (1 μM in DMSO; Sigma), doxycycline (1 µg ml^−1^ in water; Sigma), AZ5576 (0.5 µM in DMSO; MedChemExpress) and THZ531 (0.5 µM in DMSO; MedChemExpress).

### ChIP–seq

Collected mESCs were mixed with spike-in *Drosophila* S2 or HEK293 cells and immediately crosslinked with 1% formaldehyde in 1× PBS for 10 min at room temperature, followed by quenching with 125 mM glycine at 4 °C. After washing the ESCs twice with PBS, ESCs were lysed in LB1 buffer (50 mM HEPES, pH 7.9, 140 mM NaCl, 1 mM EDTA, 10% glycerol, 0.5% NP-40, 0.25% Triton X-100) for 20 min at 4 °C. Then, samples were washed in LB2 buffer (10 mM Tris–HCl, pH 8.0, 200 mM NaCl, 1 mM EDTA, 0.5 mM EGTA) for 5 min at 4 °C. Chromatin was extracted by incubation in LB3 buffer (10 mM Tris–HCl, pH 8.0, 100 mM NaCl, 1 mM EDTA, 0.5 mM EGTA, 0.1% DoC-Na, 0.5% sodium sarcosinate, 1% Triton X-100) for 10 min on ice followed by sonication for 12 min. The sonicated chromatin was spun down at 16,000*g* for 10 min at 4 °C and the supernatant was incubated with the appropriate antibody overnight at 4 °C. Antibody-bound chromatin fragments were collected by incubation with magnetic Protein A (Invitrogen, 10001D) or G DynaBeads (Invitrogen, 10003D) for 3 h at 4 °C. The beads were washed for 5 min at 4 °C with the following buffers: RIPA-150 (50 mM Tris–HCl, pH 8.0, 150 mM NaCl, 0.1% SDS, 0.5% DoC-Na, 1% NP-40), RIPA-500 (50 mM Tris–HCl, pH 8.0, 500 mM NaCl, 0.1% SDS, 1% NP-40), Li-Buffer (50 mM Tris–HCl, pH 8.0, 250 mM LiCl, 0.5% DoC-Na, 1% NP-40) and TE buffer (50 mM Tris–HCl, pH 8.0, 10 mM EDTA). After an additional wash with TE buffer, beads were resuspended in 100 μl of elution buffer (50 mM Tris–HCl, pH 7.4, 1% SDS, 1 mM EDTA) and crosslinks were reversed by incubation at 65 °C for at least 6 h. RNA and proteins were degraded by further incubations with RNase A and proteinase K, respectively. The resulting DNA was purified using ChIP DNA Clean and Concentrator kit (Zymo Research) according to the manufacturer’s protocol. Purified input and ChIP DNA were used for qPCR (Supplementary Table 2) or to prepare Illumina multiplexed sequencing libraries using NEBNext Ultra II DNA Library Prep Kit.

### Native histone ChIP–seq

For native histone ChIP–seq, 3 × 10^6^ ESCs per replicate were collected using Accutase, mixed with spike-in cells and flash-frozen upon PBS wash. The cell pellet was thawed on ice and resuspended in 150 μl of 1× PBS and mixed with 150 μl of 2× lysis buffer (100 mM Tris–HCl, pH 8.0, 0.2% Triton X-100, 0.1% DOC-Na, 10 mM CaCl_2_, 10 mM sodium butyrate) containing 0.9 μl of MNase (NEB). The cell lysate was incubated first for 20 min at 4 °C and then for 10 min at 37 °C. The MNase digestion was quenched by the addition of 30 μl of 25 mM EGTA. Afterwards, the sample was immediately placed on ice and mixed occasionally by inverting the tube for 2 min. The soluble chromatin was collected by spinning down the sample at 16,000*g* for 5 min at 4 °C and mixed with the dilution buffer (50 mM Tris–HCl, pH 8.0, 150 mM NaCl, 0.1% DOC-Na, 2.2% Triton X-100, 50 mM EDTA, 50 mM EGTA, 5 mM sodium butyrate). A total of 2% soluble fraction were taken as input and the rest was incubated overnight with the histone PTM antibody at 4 °C. The next day, magnetic Protein A (Invitrogen, 10001D) or G DynaBeads (Invitrogen, 10003D) were washed thrice in PBS and 20 μl per sample were added to collect the antibody-chromatin conjugates. Then, they were washed in the following buffers (each for 5 min at 4 °C): low salt buffer (0.1% SDS, 1% Triton X-100, 0.1% DOC-Na, 20 mM Tris–HCl, pH 8.0, 2 mM EDTA, 150 mM NaCl) (twice), high salt buffer (0.1% SDS, 1% Triton X-100, 0.1% DOC-Na, 20 mM Tris–HCl, pH 8.0, 2 mM EDTA, 360 mM NaCl), LiCl buffer (0.25 M LiCl, 1% NP-40, 1% DOC-Na, 1 mM EDTA, 10 mM Tris–HCl, pH 8.0) (twice). Finally, the samples were rinsed once in TE buffer (50 mM Tris–HCl, pH 8.0, 10 mM EDTA) and resuspended in 100 μl of elution buffer (50 mM Tris–HCl, pH 7.4, 1% SDS, 1 mM EDTA) and incubated first for 1 h at 55 °C with proteinase K and then for 30 min at 37 °C with RNase A. The resulting DNA was purified using ChIP DNA Clean and Concentrator kit (Zymo Research) according to the manufacturer’s protocol. Illumina multiplexed sequencing libraries were prepared from 1–10 ng of purified input and ChIP DNA using the NEBNext Ultra II DNA Library Prep Kit.

### Total RNA-seq

For total RNA-seq and quantitative RT–PCR (RT–qPCR) experiments, ESCs seeded on 6-well plates were directly lysed on the plate using 300μl of TRIzol reagent (Invitrogen). This lysate was either stored at −80 °C or immediately processed for RNA purification using the Direct-Zol RNA Miniprep kit (Zymo Research) according to the manufacturer’s instructions. RNA concentration was measured using Qubit 2.0 (Thermo Fisher Scientific). Total RNA of high quality was used for RT–qPCR (Supplementary Table 2) or library preparation using Illumina Stranded TruSeq Total RNA RiboZero Plus kit.

### TT-seq

TT-seq was performed as previously described with minor modifications^25^—ESCs were treated with 500 μM 4-sU (Sigma) for 5 min under regular growth conditions and lysed using TRIzol (Invitrogen) immediately afterwards. Total RNA was purified using chloroform and isopropanol precipitation and resuspended in the TE buffer. Three hundred micrograms of total RNA for each sample was spiked with a spike-in mix (Supplementary Table 3) and fragmented using Bioruptor Plus (30 s on/30 s off, one cycle, HIGH setting) and an aliquot was taken as total RNA sample. The remaining fragmented total RNA was biotinylated with Biotin-HPDP (Thermo Fisher Scientific) at room temperature for 2 h. To remove excess Biotin-HPDP, RNA was precipitated using phenol:chloroform-isoamyl alcohol (Sigma) and ethanol and resuspended in ddH_2_O. Biotinylated RNA fragments were purified using μMACS streptavidin beads and μMACS columns (Miltenyi Biotec) according to the recommendations of the manufacturer. Labeled RNA was eluted twice using 100 mM DTT and purified using Oligo Clean and Concentrator kit (Zymo Research). Both total and labeled RNA samples were treated with Turbo DNase (Invitrogen) for 30min at 37°C and purified with the Oligo Clean and Concentrator kit (Zymo Research) again. Libraries were prepared using Illumina Stranded Total RNA with RiboZero Plus according to the manufacturer’s instructions.

### PRO-seq

Cells were collected using Accutase and washed with 10 ml of ice-cold PBS. Then, the cell pellet was resuspended in 250 µl of buffer W (10 mM Tris–HCl, pH 8.0, 10 mM KCl, 250 mM sucrose, 5 mM MgCl_2_, 1 mM EGTA, 10% (vol/vol) glycerol, 0.5 mM DTT, SUPERase In 0.2 µl ml^−1^) and mixed with 10 ml of buffer P (buffer W supplemented with 0.1% (vol/vol) NP-40, 0.05% (vol/vol) Tween-20). Upon incubation for 5 min on ice, the permeabilized cells were spun down and washed with 10 ml of buffer W. The pellet was resuspended in 400 µl of buffer F (50 mM Tris–HCl, pH 8.0, 40% (vol/vol) glycerol, 5 mM MgCl_2_, 1.1 mM EDTA, 0.5 mM DTT, SUPERase In 1 µl ml^−1^) and the number of cells in each sample were counted upon diluting a 10-µl aliquot in PBS. The samples were aliquoted into DNA low-bind tubes to a cell density of 1 × 10^6^ cells per 45 µl of buffer F and snap-frozen. After thawing, the samples were mixed with permeabilized *Drosophila* S2 cells as spike-in. The mixed-permeabilized cells were used for run-on reactions containing biotin-dNTPs and sarkosyl. Purified biotinylated RNA was subjected to chemical fragmentation at 95 °C for 5 min. Enrichment of nascent RNA and library preparation were performed as previously described^52^.

### Super-resolution imaging

Live-cell PALM was carried out on mESCs expressing Halo-tagged protein of interest. A total of 100,000 ESCs were seeded the night before the experiment on 35 mm MatTek imaging dishes (P35G-1.5-20-C) that were precoated with 5 µg ml^−1^ poly-L-ornithine (Sigma, P4957; for at least 5 h) and 10 µg ml^−1^ mouse laminin (Gibco, 23017015; for at least another 5 h). On the day of the experiment, ESCs were incubated with 100 nM photoactivatable Halo ligand, PA-JF646 (a kind gift of Luke Lavis laboratory), for 10 min, washed twice with imaging medium (mESC culturing medium with phenol red-free DMEM (Gibco)), followed by a further 20-min incubation to wash out the unbound ligand. Cells were simultaneously illuminated with low intensity near ultraviolet light (405 nm), which was gradually increased from 0.1% to 10% over the time of the acquisition, for photoactivation of the Halo ligand for fluorescence detection with an exposure time of 50 ms and images were acquired for 10,000 frames.

Image analysis was performed using the qSR tool^53^. DBSCAN embedded in the qSR software was used to define clusters with a minimum of 25 nearby localizations within 100 nm. Transient clusters were manually marked and verified for calculating the cluster lifetime.

### ChIP–seq analysis

ChIP–seq data were processed using the default parameters of snakePipes (v2.5.1) DNA-mapping and ChIP–seq pipelines with ‘--trim --dedup --mapq 3 --properPairs’ options^54^. The data were mapped to the mm10 genome for standard analysis and to a hybrid genome consisting of mm10 and spike-in genome builds for spike-in normalization. For data visualization, replicates with a Pearson correlation *R* > 0.9 are merged. Heatmaps and metagene plots have been generated using deepTools^54,55^. To perform differential binding analysis, R Diffbind package was used^56^. The genome snapshots were generated using pyGenomeTracks^57^.

### TT-seq analysis

TT-seq data analysis was performed as previously described^58^. The data were processed through the snakePipes mRNA-seq pipeline (modified version of v2.1.2), similar to RNA-seq analysis^54^. Adaptors and low-quality bases (<Q20) were removed using Cutadapt (v2.8)^59^. For all of the samples, reads were then mapped to the reference genome (mm10) and a custom genome index containing spike-in sequences with STAR (v2.7.4a)^60^. Then, reads per gene were counted using featureCounts (v2.0.0)^61^. The gene-level counts obtained from featureCounts were then used for differential expression analysis using DESeq2 (v1.26.0), with size factors calculated from spike-in counts, after filtering out lowly expressed genes^62^. For transcriptional rate calculations, read counts for all genes were obtained from each corresponding labeled and unlabeled TT-seq sample. To estimate the rates of RNA degradation and synthesis, we used a statistical model that was described previously^25^.

### PRO-seq analysis

Dual, 6-nucleotide Unique Molecular Identifiers (UMIs) were extracted from read pairs using UMI tools (v1.1.2) and adaptor sequences were removed using Cutadapt (v3.5)^59,63^. After trimming, reads shorter than 26 nucleotides were discarded. Then, the paired-end reads were mapped to a hybrid genome, which includes the spike-in genome build, dm6 and the primary genome build, mm10, using bwa-mem2 (v2.3-0) with ‘-k 19’ setting^64^. Alignments were coordinate-sorted and indexed using SAMtools (v1.22.1) and only properly paired reads with a mapping quality greater than 20 were retained^65^.

The BAM files were split by the genome using Sambamba (v0.8.1), independently deduplicated using UMI tools and coordinate-sorted^63,66^. Then, the BAM files were converted into BEDPE format using BEDtools and strand-aware, single-nucleotide positions corresponding to 3′ and 5′ ends of nascent RNA were extracted from the first mapped position of R1 and R2 reads, respectively^67^. Plus and minus strands were swapped to generate strand-specific single-nucleotide coverage tracks using deepTools (v3.5.6)^68^. Spike-in normalization factors were calculated for each sample by dividing the number of reads mapped to the spike-in *dm6* genome by the number of *dm6* reads in the first replicate of DMSO control. Replicates were merged for data visualization.

### Statistics and reproducibility

All statistical analyses were performed using GraphPad Prism (v10.2.0) or R (v4.1.3). No statistical method was used to predetermine sample size. Sample size, number of replicates, statistical tests and definition of error bars were stated in the legends of Figs. 1–7 and Extended Data Figs. 1–8. No data were excluded from data analysis. The experiments were not randomized. The investigators were not blinded to allocation during experiments and outcome assessment. Western blot, live-cell imaging, immunofluorescence and super-resolution microscopy experiments were independently repeated with similar results at least twice.

### Reporting summary

Further information on research design is available in the Nature Portfolio Reporting Summary linked to this article.

## Online content

Any methods, additional references, Nature Portfolio reporting summaries, source data, extended data, supplementary information, acknowledgements, peer review information; details of author contributions and competing interests; and statements of data and code availability are available at 10.1038/s41588-026-02533-x.

## Supplementary information

Supplementary Information Supplementary Methods and Notes 1–5.

Reporting Summary

Supplementary Table 1 BRD2 interactome dataset.

Supplementary Table 2 Oligonucleotide sequences usedin this study.

Supplementary Table 3 List of ERCC spike-ins for TT-seq.

Supplementary Video 1 Live cell imaging of BRD2-mEGFP in mESCs upon flavopiridol treatment.

## Source data

Source Data for Fig. 1 Unprocessed western blots.

Source Data for Fig. 2 Unprocessed western blots.

Source Data for Fig. 4 Statistical source data.

Source Data for Fig. 5 Statistical source data.

Source Data for Fig. 6 Statistical source data.

Source Data for Fig. 7 Statistical source data.

Source Data for Extended Data Fig. 1 Statistical source data.

Source Data for Extended Data Fig. 1 Unprocessed western blots.

Source Data for Extended Data Fig. 2 Statistical source data.

Source Data for Extended Data Fig. 2 Unprocessed western blots.

Source Data for Extended Data Fig. 3 Unprocessed western blots.

Source Data for Extended Data Fig. 5 Statistical source data.

Source Data for Extended Data Fig. 6 Statistical source data.

Source Data for Extended Data Fig. 6 Unprocessed western blots.

Source Data for Extended Data Fig. 7 Statistical source data.

Source Data for Extended Data Fig. 7 Unprocessed western blots.

Source Data for Extended Data Fig. 8 Statistical source data.

Source Data for Extended Data Fig. 8 Unprocessed western blots.

## Data Availability

All the NGS datasets and differential expression analysis tables generated in this study have been deposited to the Gene Expression Omnibus under the accession GSE271982. The mass spectrometry proteomics data have been deposited to the ProteomeXchange Consortium through the PRIDE partner repository with the dataset identifier PXD072982. Source data are provided with this paper.

## Code availability

We have made use of the publicly available software, tools and R scripts for data analysis. Scripts for the analysis of RNA synthesis rates from TT-seq data are available on Zenodo (10.5281/zenodo.18403045)^69^.
