# Supplementary material for: Histone acetylation-dependent clustering of BRD2 instructs transcription dynamics
Source: Nat Genet. 2026 Apr 9;58(4):854–68. doi: 10.1038/s41588-026-02533-x (PMC13083254; doi:10.1038/s41588-026-02533-x)
Supplement: Supplementary file 1 — Supplementary Methods and Notes 1–5. [file 41588_2026_2533_MOESM1_ESM.pdf]

nature genetics

Supplementary information https://doi.org/10.1038/s41588-026-02533-x

Histone acetylation-dependent clustering of BRD2 instructs transcription dynamics

In the format provided by the authors and unedited

Histone acetylation-dependent clustering of BRD2 instructs transcription dynamics

Niyazi Umut Erdogdu[1,2], Sukanya Guhathakurta[1,3], Ronald Oellers[1,2], Maria Shvedunova[1], Jose A. Morin[1], Eric M. Patrick[1], Janine Seyfferth[1], Ward Deboutte[1], Alejandro Gomez-Auli[1], Gerhard Mittler[1], Ibrahim I. Cissé[1,3], Asifa Akhtar[1,3 #]

[1] Max Planck Institute of Immunobiology and Epigenetics, Freiburg, Germany

[2] Faculty of Biology, University of Freiburg, Freiburg, Germany

[3] CIBSS - Centre for Integrative Biological Signalling Studies, University of Freiburg

[#] Corresponding author. E-mail: akhtar@ie-freiburg.mpg.de

Supplementary Information

Supplementary Movie 1: Live cell imaging of BRD2-mEGFP in mESCs upon flavopiridol treatment

Supplementary Table 1: BRD2 interactome dataset

Supplementary Table 2: Oligonucleotide sequences used in this study

Supplementary Table 3: List of ERCC spike-ins for TT-Seq

Supplementary Notes 1-5

Supplementary Methods

Supplementary References

SUPPLEMENTARY NOTES

Supplementary Note 1

Despite overlapping genome-wide chromatin binding of BET proteins, only BRD2 predominantly responds to the loss of MOF-mediated H4ac. This was further corroborated by a recent proteomic profiling study of chromatin readers suggesting that BET proteins preferentially bind to H4ac rather than H3ac [1]. Considering the dramatic loss of BRD2 chromatin binding upon MOF depletion, it is very likely that the specificity of BRD2's bromodomain modules may more strongly depend on H4K16ac and its combination with other H4ac marks in comparison to BRD3 and BRD4 [2,3]. Since these proteins almost exclusively associate with actively transcribed regions which are enriched for both H4ac and H3ac, their combination might be potentially maintaining the chromatin association of BRD3 and BRD4 due to an avidity effect. Accordingly, their loss of chromatin binding might have required the single or combined depletion of other HATs.

Supplementary Note 2

Even though we majorly focused on the functional relationship between BET proteins in transcription regulation, they also have been proposed to have distinct roles in the regulation of genome architecture: It has been recently demonstrated that BRD2 contributes to the compartmentalization of active chromatin upon cohesin loss in an antagonistic manner with BRD4 [4]. Nevertheless, the impact of BRD2 depletion alone on genome compartmentalization was not prominent (Extended Data Figure 3e-g) [4,5]. BRD2 has also been suggested to be involved in the formation of transcriptional boundaries in collaboration with CTCF and cohesin [6,7]. Interestingly, BRD4 has been shown to orchestrate genome folding during neural crest differentiation by its interaction with cohesin cofactor NIPBL [8]. In this respect, the dynamic interplay between BET proteins and cohesin in genome folding throughout physiological and potentially pathological cellular states remains to be further studied.

Supplementary Note 3

While mapping of 3'-ends of PRO-Seq reads allows us to infer the location of RNA Pol II at single-nucleotide resolution, 5' end mapping can be used to approximate

initiation sites. To quantitatively analyze transcription from actively transcribed promoters at their TSS and infer an estimation of transcription initiation, we first compiled a list of transcription start regions (TSRs) using PRO-Seq data and a bioinformatics tool called tsrPicker [9]. We have further subsetted these TSRs to identify the most dominant TSR per transcription unit.

Supplementary Note 4

BRD2 foci were observed in untagged mESCs and NIH 3T3 cells following flavopiridol treatment using an antibody against BRD2. The relatively low signal-to-noise ratio of these foci, compared with detection using an anti-V5 antibody in BRD2 degron mESCs expressing BRD2-mAID-V5, could potentially be attributed to differences in antibody affinity (Extended Data Fig. 6l).

Supplementary Note 5

Our *in vitro* phase-separation experiments using BRD2-IDR also demonstrate that increasing RNA concentrations can efficiently disrupt BRD2 condensates, suggesting that BRD2 chromatin association in cells is favored under conditions of low nascent RNA output, such as during elongation blockade. Considering the prominent role of BRD4 in the control of nascent RNA synthesis and the genome-wide overlap of BRD2 and BRD4 chromatin binding, our findings imply that BRD2 acts as a sensor of prolonged RNA Pol II pausing and nascent transcription. Another interesting aspect to consider is that inhibition of pause release also affects the dynamics of RNA Pol II clusters by increasing their half life [10]. It is very likely that many other accessory factors and coactivators for transcription also enrich the chromatin and collectively create a micro-environment poised for transcription. However, the exact mechanism by which increased chromatin binding of these transcription coactivators is achieved upon inhibition of pause release remains to be further studied.

SUPPLEMENTARY METHODS

Generation of stable cell lines with inducible expression

DNA sequences encoding for different BRD2 variants were first cloned into an entry vector (a gift from Knut Woltjen, Addgene #120359) by restriction digestion. Then, the inserts were moved into the PiggyBac (PB) destination vector PB-TAG-ERN (a gift from Knut Woltjen, Addgene # 80476) by Gateway recombination using LR Clonase II (Invitrogen # 11791020). 0.5 µg PB vector was co-transfected with 0.2 µg of PB transposase vector (System BIosciences, PB210PA-1) into mESCs using Lipofectamine 2000 (Invitrogen, # 11668027). 48 h after transfection, cells were selected for positive integration using G418 (Gibco, # 10131035). Positive clones were picked using pipette tips and expression of the inserts were further investigated by FACS or western blot upon induction with doxycycline.

Immunoblotting

Cellular pellet was resuspended and lysed in SDS loading dye (2X ROTI-Load , Carl Roth). Samples were boiled at 95°C for 5 minutes, sonicated using the Branson sonifier 250 (40% duty cycle, 1.5 output, 10 pulses) and boiled again for 5 minutes. SDS-PAGE was performed by running samples on NuPAGE Bis-Tris 4-12 % gradient gels (Invitrogen #NP0321PK2) using MOPS (Novex #NP0001, for detection of proteins with molecular weight higher than 50 KDa) or MES buffer (for detection of proteins with molecular weight lower than 50 KDa, for example histones).

For detection of proteins with molecular weight lower than 30 kDa (for example, the histones), proteins were wet-transferred to a 0.22 µm PVDF membrane, otherwise a 0.45 µm membrane was used. Following the transfer, the membrane was blocked with 5% skimmed milk in 0.3% Tween-PBS for 1 hour at RT and incubated with relevant primary antibodies overnight. After washing thrice for 5 minutes each with 0.3% Tween-PBS, membranes were incubated with HRP-conjugated secondary antibodies for 1.5 hours at RT. The membrane was washed thrice for 5 minutes each with 0.3% Tween-PBS and finally developed with Lumi light enhanced chemiluminescence substrate (Roche #12015196001) and imaged using BioRad ChemiDoc system.

Confocal microscopy

For immunofluorescence, mESCs grown on coverslips to a confluency of 70-80% were fixed with 4% formaldehyde, diluted in PBS, at RT for 15 minutes and permeabilized with 0.2% Triton X-100-PBS for 10 minutes. They were then blocked with 4 % BSA, prepared in 0.05% Triton X-100-PBS for 1 hour and then incubated overnight with primary antibodies in the above blocking buffer at 4°C. Next day, the cells were washed thrice at RT, 10 mins each, with 0.05% Triton X-100-PBS and then incubated with fluorophore-conjugated secondary antibodies for 2 hours at RT in the dark. Then they were washed twice, 10 minutes each, with 0.05% Triton X-100-PBS and then counterstained with 20 μM Hoechst 33342 (Invitrogen) in PBS for 10 minutes. The coverslips were mounted on glass slides with using FluoroGel (#GTX2814) after a final PBS wash of 10 minutes.

For steady and time-lapse live cell imaging, ESCs were grown on eight-well glass bottom Ibidi chambers (#80827) 6-8 hours before the start of the experiment. Experiments were performed using inverted confocal laser scanning microscope Zeiss LSM 880 equipped with an Airyscan detector. The laser power was set to a maximum of 1% to minimize photobleaching and phototoxicity. Images were acquired at different focal planes with appropriate Z-stack settings to cover the width of the nucleus.

Fluorescence Recovery After Photobleaching (FRAP) experiments were performed as follows: 1-3 equal sized regions of interest in a cell were selectively bleached with 100% laser power and images were collected each 2 seconds. Fluorescence intensity of the regions of interest was calculated and corrected against the mean of 2 same sized regions containing unbleached clusters for the corresponding field of view. Image acquisition and analysis were performed using Zen2 Blue Software (v. 3.1 and 3.2, Zeiss).

Protein purification and *in vitro* assays

Design of the construct, purification process of the protein and *in vitro* droplet assay was performed as previously described with minor modifications [11]. BRD2-IDR was amplified from cDNA and the eGFP tag was incorporated at its 5' end. The recombinant BRD2-IDR-eGFP was cloned into a pET41b expression vector (a kind

gift of Erinc Hallacli) and transformed into BL21(DE3)LysS bacterial strain (Invitrogen). Cells were grown in LB containing kanamycin and chloramphenicol at 37°C till the OD of 0.6-0.7 and then 1 mM IPTG was added to allow protein expression at 18°C overnight. The bacterial culture was collected by centrifugation and the pellet was resuspended in Buffer A (50mM Tris pH7.5, 500 mM NaCl) containing 10mM imidazole, cOmplete protease inhibitors (Roche, 11873580001) and sonicated (10 cycles of 15 seconds on, 60 seconds off). The lysate was clarified by centrifugation at 12,000g for 30 minutes at 4°C and added to Ni-NTA agarose slurry (Invitrogen, R901-15), pre-equilibrated with 10X volumes of Buffer A. The recombinant protein was allowed to bind to the column through its His-tag at 4°C for 1.5 hours, with gentle rotation. The agarose slurry was poured into a column, packed, and washed with 15x volumes of Buffer A containing 10mM imidazole. Protein was eluted in multiple fractions with Buffer A with 250mM imidazole. Fractions of elutions containing protein as judged by coomassie stained gel were combined and dialyzed against Buffer B (50mM Tris-HCl pH7.5, 500 mM NaCl, 10% glycerol, 1mM DTT).

Recombinant BRD2-IDR-eGFP was concentrated and desalted to an appropriate protein and salt concentration using Amicon Ultra centrifugal filters (30K MWCO, Millipore). Solutions containing varying concentrations of the recombinant protein, salt, RNA or ATP were prepared, incubated for 30 minutes at RT and loaded onto glass slides and mounted with cover slips. Slides were then imaged using an inverted confocal laser scanning microscope Zeiss LSM 880 equipped with an Airyscan detector.

BRD2 interactome

In order to minimize the saturation of MS peaks with antibody peptides used for co-immunoprecipation, antibodies were crosslinked to magnetic DynaBeads Protein G (Invitrogen, #10003D) using DMP: Per IP, 50 µL of Dynabeads was washed twice with 500 µL of 0.1 M phosphate buffer (pH 8.0) by inverting and gentle vortexing. Then, the beads were first incubated with 6 µg of the antibody in phosphate buffer (pH 8.0) in a total volume of 100 µL for 30 minutes at 4 °C. Afterwards, Tween-20 was added to 0.1 % and the sample was further incubated at 4 °C overnight. The next day, the beads were first washed thrice in 500 µL of phosphate buffer and then

twice in 500 µL of 0.2 M triethanolamine. The antibody was crosslinked to the beads by incubation with 500 µL DMP (6.5 mg/mL in 0.2 M triethanolamine) for 45 minutes at room temperature. The crosslinked beads were rinsed once with 500 µL of 0.1 M ethanolamine and then incubated in 0.1 M ethanolamine for 30 minutes at room temperature. After washing thrice with 500 µL of PBS, the excess non-crosslinked antibody was washed away by incubating the beads in 500 µL of 0.1 M Glycine-HCl (pH 2.5) by inverting and gentle vortexing. The beads were then washed twice with 1X PBS and finally resuspended in 100 µL of 1X PBS supplemented with 0.1 % Tween-20 and 0.02 % Na-Azide. The beads were stored at 4 °C until use.

1 x 10^7 cells were harvested per replicate using accutase and flash-frozen. The cell pellet was thawed on ice and lysed in 2 mL of Buffer A (10 mM HEPES-KOH, pH 7.9, 5 mM $MgCl_2$, 10 mM KCl, 1 mM DTT, 0.1 % NP-40) by rotating for 10 minutes at 4 °C. The nuclei were collected by spinning down the sample for 10 min at 2000 rpm at 4 °C. The nuclear pellet was resuspended in 100 µL of buffer B (25 mM HEPES-KOH, pH 7.5, 150 mM KCl, 10 % Glycerol (v/v), 12.5 mM $MgCl_2$, 0.2 % NP-40, 5 mM $CaCl_2$) and kept on ice for 10 minutes. Then, 2 µL of MNase (NEB) was added and the sample was incubated for 10 min at 37 °C in a thermomixer at 1000 rpm. MNase digestion was quenched by addition of 0.73 µL of 330 mM EGTA. The soluble nuclear fraction was collected by spinning down the samples at 16000 g for 10 min at 4 °C. The volume of the samples brought to 500 µL using IP buffer (25 mM HEPES-KOH, pH 7.5, 150 mM KCl, 10 % Glycerol (v/v), 12.5 mM $MgCl_2$, 0.2 % NP-40, 31.25 mM EDTA, 31.25 mM EGTA). The crosslinked beads were washed thrice in IP buffer and then incubated with the samples overnight at 4 °C. The next day, beads were washed three times with 500 µL of IP buffer and three times with 500 µL of WB2 buffer (25 mM HEPES-KOH, pH 7.5, 150 mM KCl, 5 % Glycerol (v/v), 5 mM $MgCl_2$, 0.02 % Rapigest-MS).

Mass spectrometry sample preparation

Beads were resuspended in 50 µL of 2TU buffer (2 M urea, 100 mM Tris-HCl, pH 8.0, 0.04% Rapigest) and proteins were reduced by addition of TCEP (*ad* 10 mM, 5 minutes at 25 °C), followed by alkylation with CAA (*ad* 40 mM, 5 minutes at 25 °C). Immunoprecipitated proteins were digested with 0.2 µg trypsin (Promega) and 0.2 µg Lys-C (Wako) for 90 minutes at 25 °C. Afterwards, digested peptides were

transferred into a fresh tube. Magnetic beads were further incubated with 50 mM ammonium bicarbonate buffer for 10 minutes at 25 °C and the extracted peptides were collected and pooled. The peptide sample pool was further digested with 0.28 µg of trypsin at 25 °C overnight. Digestion was stopped by addition of 10 µL of 10 % TFA, and further mixed with 50 µL of Stage A buffer (0.5 % acetic acid, 0.02 % HFBA). After spinning down for 10 min at 14,000xg, the samples were loaded onto in-house packed C18 StageTips (2 disc layers) for desalting and clean-up as described [12,13].

Liquid chromatography-mass spectrometry

Samples were measured on an Exploris 480 MS interfaced with a EASY-nLC 1200 liquid chromatography system (both ThermoFisher Scientific) using a 60-minute gradient, and a top 20 DDA method, as previously described [13]. Samples were injected thrice and measured in data-dependent mode following a “fast”, “sensitive” (both as in [13]), and an “ultra sensitive” method which included an isolation window of 1.8 m/z and MS2 resolution of 30000, and a maximum injection time of 180 ms.

Mass spectrometry data analysis

Obtained RAW files were processed using MaxQuant (v1.6.14.0)[14] employing default parameters (with details listed below), against a UniProt *Mus musculus* database containing Swiss-Prot, TrEMBL, and isoforms sequences (63639 entries, downloaded 24.01.2022) plus an in-house curated contaminants database based on the default MaxQuant contaminant list. Trypsin/P was set as enzyme allowing up to 2 missed cleavages. Modifications included fixed cysteine carbamidomethylation, and variable oxidation (M), acetylation (Protein N-termini), and deamidation (NQ). Relative quantification was calculated using MaxLFQ. Match between-runs was activated (time window 0.5 min), and data processing was set up so that matching only occurred within each sample group.

The obtained *proteinGroups.txt* was further processed with R (v4.2.2) / RStudio (v2023.3.1.446) using ProteomeR, an in-house developed R package assembled from extensive R functions [15]. Briefly, “reversed”, “only identified by site”, and “contaminants” entries were removed. Only proteins with at least 2 valid values in any condition were considered for further processing. Missing values were imputed if

consistently missing in a condition or missing in more than 50 % of the replicates using a left-censor strategy (width = 0.3, shift = 1.8)[16] and the rest using the *missForest* approach[17]. For hypothesis testing, a linear model was fitted using *limma* (trend=TRUE)[18,19]. Obtained p-values were adjusted for multiple hypothesis testing using the Benjamini-Hochberg procedure [20]. Differentially abundant protein groups were considered by an adjusted p-value ≤ 0.05.

MicroC

MicroC was performed as previously described with some modifications [21]: Harvested cells were collected in 10 % FBS in PBS and first crosslinked in 2 % formaldehyde for 10 minutes at RT. Upon quenching with 0.128 M glycine for 5 minutes at RT and for 10 minutes on ice, cells were washed with ice-cold PBS and crosslinked with 3 mM DSG in PBS for 40 minutes at RT. Upon quenching with 0.4 M glycine for 5 minutes at RT, cells were washed again with ice-cold PBS and lysed in 100 µL of MB #1 (10 mM Tris-HCl, pH 7.5, 50 mM NaCl, 5 mM $MgCl_2$, 1 mM $CaCl_2$, 0.2 % NP-40, 1x Roche cOmplete EDTA-free) / 1 x 10^6 cells. Upon incubation on ice for 20 minutes, the sample was washed with ice-cold MB #1 and the chromatin was fragmented in MB #1 using MNase (Worthington Biochem #LS004798) at 37 °C for 9 minutes. The digestion was stopped by the addition of 4 mM EGTA. Upon incubation for 10 minutes at 65 °C, the sample was washed twice with ice-cold MB #2 (10 mM Tris-HCl, pH 7.5, 50 mM NaCl, 10 mM $MgCl_2$).

To generate blunt-ended, end-repaired DNA fragments, samples were first treated with T4 PNK (NEB) in NE Buffer 2.1 base containing 2 mM ATP and 5 mM DTT at 37 °C for 15 minutes. Then, the samples were treated with Klenow fragment (NEB) at 37 °C for 15 minutes. End-labeling was performed by treating the samples with a mixture of 66 mM biotin-dATP, biotin-dCTP, biotin-dTTP and biotin-dGTP, 1x BSA and 0.5X T4 DNA ligase buffer (NEB) for 45 minutes at 25 °C. The reaction was stopped by heat-inactivation at 65 °C for 20 minutes upon addition of 30 mM EDTA. Samples were washed once with ice-cold MB #3 (50 mM Tris-HCl, pH 7.5, 10 mM $MgCl_2$). Upon washing the samples with 1X T4 DNA Ligase buffer (NEB), the pellet was resuspended in 500 µL of reaction mix for proximity-ligation (1x DNA Ligase Buffer (NEB), 1X BSA, T4 DNA Ligase (NEB)) and incubated at RT for 3 h. The

chromatin pellet was then treated with Exonuclease III (NEB) at 37 °C for 15 minutes to remove biotin from unligated ends.

Crosslinks were reversed by treating the samples with Proteinase K and RNAse A at 65 °C overnight. Dinucleosomal DNA was purified by phenol:chloroform:isoamyl alcohol extraction followed by gel purification of 220-400 bp sized DNA from 2 % agarose gel. The biotin-bound, ligated DNA-contact fragments were pulled down using Dynabeads MyOne Streptavidin C1 magnetic beads (Invitrogen). Libraries were prepared on beads using NEBNext Ultra II DNA Library Prep kit.

MicroC analysis

MicroC data was analyzed with snakePipes (v. 3.2.0), using the makePairs function [22]. Briefly, fastq files were trimmed using fastp (v. 0.23.4) and aligned to the mm10 reference genome using bwa (v. 0.7.19-r1273) [23,24]. Pairtools (v. 1.1) was subsequently used to extract, deduplicate and index valid interaction pairs [25]. Single replicates had, on average, more than 100 x 10^6 valid, unique interactions and conditions were run in triplicates. Cooler (v. 0.10.3) was subsequently used to convert the pair files into balanced cool files containing resolutions at 5 kB, 10 kB, 15 kB and 20 kB to 120 kB in increments of 20 kB [26]. Compartmentalization analysis was performed by using cooltools (v. 0.7.1) with cool files at 100 kB resolution [27]. The eigs_cis function within cooltools was used to calculate the compartment scores with three eigenvectors informed by the genomic GC content. Differences between compartment scores were assessed by using a two-sample Kolmogorov-Smirnov test with the ks_2samp function implemented in SciPy (v. 1.15.1) [28]. Pile-up and saddleplot analysis was performed using coolpuppy (v. 1.1.0), using the cooler files at 15kb resolution [29].

Total RNA-Seq analysis

Total RNA-Seq was processed using default parameters of SnakePipes (v. 2.5.1) noncoding-RNA-seq pipeline with "--trim" option [22]. The data was mapped to the mm10 genome. Downstream analysis and visualization was performed using featureCounts (v.2.0.0), and R packages DESeq2 (v.1.34.0), ggplot2 (v.3.3.5) [30–32]. Volcano plots were produced using EnhancedVolcano package in R

(github.com/kevinblighe/EnhancedVolcano). For time-course experiments, unless stated otherwise, the 'untreated' condition was always taken as reference.

TSR calling and annotation

PRO-Seq bam files from all deduplicated DMSO control replicates were merged separately for host and spike-in genomes using samtools (v. 1.22.1) and then converted to BED format using bedtools (v. 2.31.1) [33,34]. TSRs were called using PolTools tsrFinder (v. 1.0.7) using a window size of 20 bp and a minimum sequence depth of 17 and 50 reads for spike-in and host samples, respectively [9]. Gene-wise TSR annotation was performed following truQuant strategy in PolTools: For each protein-coding gene, a strand-aware search window was defined from 1 kB upstream of the annotated TSS to the most downstream in-frame start codon. Within this window, the TSR with the highest 5'-end count was selected as the gene's maxTSR and recentered to an 11-bp interval around the average TSS position reported by tsrFinder.

Differential TSR analysis

Gene-level TSR activity was quantified from 5'-end profiles using featureCounts (Subread) with paired-end, primary, strand-specific settings and mapping quality filtering (-p -B -C -Q 10 –primary -s 1) yielding gene-wise 5'-end counts over maxTSRs for both host and spike-in datasets [31]. TSRs with fewer than 10 5'-end counts over their maxTSR were removed to exclude lowly expressed regions prior to differential analysis. Differential TSR analysis was performed using DESeq2, with an FDR< 0.05 and absolute fold change cut-off ≥ 1.5 [30]. For this analysis, sample-wise size factors were estimated from spike-in TSR counts by median-ratio normalization using DESeq2.

Elongation index Calculation

For each condition, an elongation index was calculated for all transcribed mRNAs between 5 to 200 kbp in length, as previously described with some modifications [35,36]: Spike-in normalized mean PRO-Seq and TT-Seq reads were added up in 100 nucleotide bins downstream of their TSSs using deeptools computeMatrix [37]. The elongation index was calculated by dividing TT-Seq coverage by PRO-Seq coverage

in these windows. The first two bins from TSS were excluded from the analysis and the bins which contain TES or are located downstream of TES were set to zero.

SUPPLEMENTARY REFERENCES

1. Lukauskas, S. *et al.* Decoding chromatin states by proteomic profiling of nucleosome readers. *Nature* **627**, 671–679 (2024).
2. LeRoy, G., Rickards, B. & Flint, S. J. The double bromodomain proteins Brd2 and Brd3 couple histone acetylation to transcription. *Mol. Cell* **30**, 51–60 (2008).
3. Umehara, T. *et al.* Structural implications for K5/K12-di-acetylated histone H4 recognition by the second bromodomain of BRD2. *FEBS Lett* **584**, 3901–3908 (2010).
4. Xie, L. *et al.* BRD2 compartmentalizes the accessible genome. *Nat Genet* **54**, 481–491 (2022).
5. Hinojosa-Gonzalez, L., Turner, J. L., Sasaki, T., Ay, F. & Gilbert, D. M. Brd2 is dispensable for genome compartmentalization and replication timing. *bioRxiv* (2023) doi:10.1101/2023.11.17.567572.
6. Hsu, S. C. *et al.* The BET Protein BRD2 Cooperates with CTCF to Enforce Transcriptional and Architectural Boundaries. *Mol Cell* **66**, 102–116.e7 (2017).
7. Cheung, K. L. *et al.* Distinct Roles of Brd2 and Brd4 in Potentiating the Transcriptional Program for Th17 Cell Differentiation. *Mol Cell* **65**, 1068–1080.e5 (2017).
8. Linares-Saldana, R. *et al.* BRD4 orchestrates genome folding to promote neural crest differentiation. *Nat Genet* **53**, 1480–1492 (2021).
9. Santana, J. F., Collins, G. S., Parida, M., Luse, D. S. & Price, D. H. Differential dependencies of human RNA polymerase II promoters on TBP, TAF1, TFIIB and XPB. *Nucleic Acids Res* **50**, 9127–9148 (2022).
10. Cho, W.-K. *et al.* Super-resolution imaging of fluorescently labeled, endogenous RNA Polymerase II in living cells with CRISPR/Cas9-mediated gene editing. *Sci*

Rep **6**, 35949 (2016).

11. Sabari, B. R. *et al.* Coactivator condensation at super-enhancers links phase separation and gene control. *Science* **361**, (2018).
12. Rappsilber, J., Mann, M. & Ishihama, Y. Protocol for micro-purification, enrichment, pre-fractionation and storage of peptides for proteomics using StageTips. *Nat Protoc* **2**, 1896–1906 (2007).
13. Lee, C. *et al.* Light-induced targeting enables proteomics on endogenous condensates. *Cell* **187**, 7079–7090.e17 (2024).
14. Cox, J. & Mann, M. MaxQuant enables high peptide identification rates, individualized p.p.b.-range mass accuracies and proteome-wide protein quantification. *Nat Biotechnol* **26**, 1367–1372 (2008).
15. Gomez-Auli, A. *et al.* The secreted inhibitor of invasive cell growth CREG1 is negatively regulated by cathepsin proteases. *Cell Mol Life Sci* **78**, 733–755 (2021).
16. Keilhauer, E. C., Hein, M. Y. & Mann, M. Accurate protein complex retrieval by affinity enrichment mass spectrometry (AE-MS) rather than affinity purification mass spectrometry (AP-MS). *Mol Cell Proteomics* **14**, 120–135 (2015).
17. Stekhoven, D. J. & Bühlmann, P. MissForest--non-parametric missing value imputation for mixed-type data. *Bioinformatics* **28**, 112–118 (2012).
18. Ritchie, M. E. *et al.* limma powers differential expression analyses for RNA-sequencing and microarray studies. *Nucleic Acids Res* **43**, e47 (2015).
19. Phipson, B., Lee, S., Majewski, I. J., Alexander, W. S. & Smyth, G. K. ROBUST HYPERPARAMETER ESTIMATION PROTECTS AGAINST HYPERVARIABLE GENES AND IMPROVES POWER TO DETECT DIFFERENTIAL EXPRESSION. *Ann Appl Stat* **10**, 946–963 (2016).

20. Benjamini, Y. & Hochberg, Y. Controlling the false discovery rate: A practical and powerful approach to multiple testing. *J. R. Stat. Soc. Series B Stat. Methodol.* **57**, 289–300 (1995).
21. Goel, V. Y., Huseyin, M. K. & Hansen, A. S. Region Capture Micro-C reveals coalescence of enhancers and promoters into nested microcompartments. *Nat Genet* **55**, 1048–1056 (2023).
22. Bhardwaj, V. *et al.* snakePipes: facilitating flexible, scalable and integrative epigenomic analysis. *Bioinformatics* **35**, 4757–4759 (2019).
23. Chen, S. Ultrafast one-pass FASTQ data preprocessing, quality control, and deduplication using fastp. *Imeta* **2**, e107 (2023).
24. Li, H. & Durbin, R. Fast and accurate short read alignment with Burrows-Wheeler transform. *Bioinformatics* **25**, 1754–1760 (2009).
25. Open2C *et al.* Pairtools: from sequencing data to chromosome contacts. *bioRxiv* (2023) doi:10.1101/2023.02.13.528389.
26. Abdennur, N. & Mirny, L. A. Cooler: scalable storage for Hi-C data and other genomically labeled arrays. *Bioinformatics* **36**, 311–316 (2020).
27. Open2C *et al.* Cooltools: Enabling high-resolution Hi-C analysis in Python. *PLoS Comput Biol* **20**, e1012067 (2024).
28. Virtanen, P. *et al.* SciPy 1.0: fundamental algorithms for scientific computing in Python. *Nat Methods* **17**, 261–272 (2020).
29. Flyamer, I. M., Illingworth, R. S. & Bickmore, W. A. Coolpup.py: versatile pile-up analysis of Hi-C data. *Bioinformatics* **36**, 2980–2985 (2020).
30. Love, M. I., Huber, W. & Anders, S. Moderated estimation of fold change and dispersion for RNA-seq data with DESeq2. *Genome Biol.* **15**, 550 (2014).
31. Liao, Y., Smyth, G. K. & Shi, W. featureCounts: an efficient general purpose

program for assigning sequence reads to genomic features. *Bioinformatics* **30**, 923–930 (2014).

32. Wickham, H. *ggplot2: Elegant Graphics for Data Analysis*. (Springer Science & Business Media, 2009).
33. Danecek, P. *et al.* Twelve years of SAMtools and BCFtools. *Gigascience* **10**, (2021).
34. Quinlan, A. R. & Hall, I. M. BEDTools: a flexible suite of utilities for comparing genomic features. *Bioinformatics* **26**, 841–842 (2010).
35. Stein, C. B. *et al.* Integrator endonuclease drives promoter-proximal termination at all RNA polymerase II-transcribed loci. *Mol Cell* **82**, 4232–4245.e11 (2022).
36. Žumer, K. *et al.* Two distinct mechanisms of RNA polymerase II elongation stimulation in vivo. *Mol Cell* **81**, 3096–3109.e8 (2021).
37. Ramírez, F. *et al.* deepTools2: a next generation web server for deep-sequencing data analysis. *Nucleic Acids Res* **44**, W160–5 (2016).
