# Supplementary figures and images for: Histone acetylation-dependent clustering of BRD2 instructs transcription dynamics
Source: Nat Genet. 2026 Apr 9;58(4):854–68. doi: 10.1038/s41588-026-02533-x (PMC13083254; doi:10.1038/s41588-026-02533-x)

Fig. 1

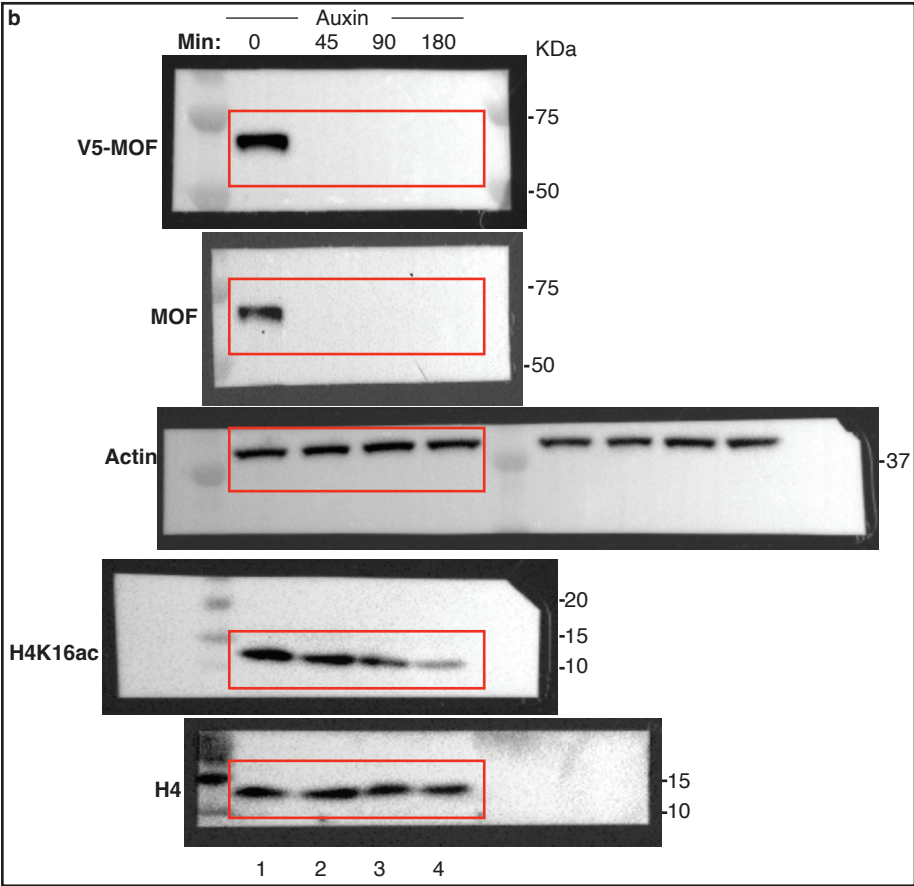

Supplement: Supplementary file 7 — Unprocessed western blots. [file 41588_2026_2533_MOESM7_ESM.pdf]

Fig. 2

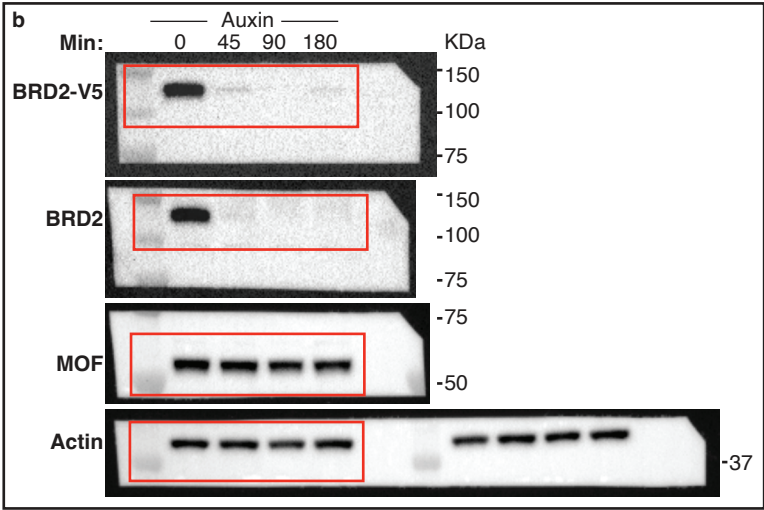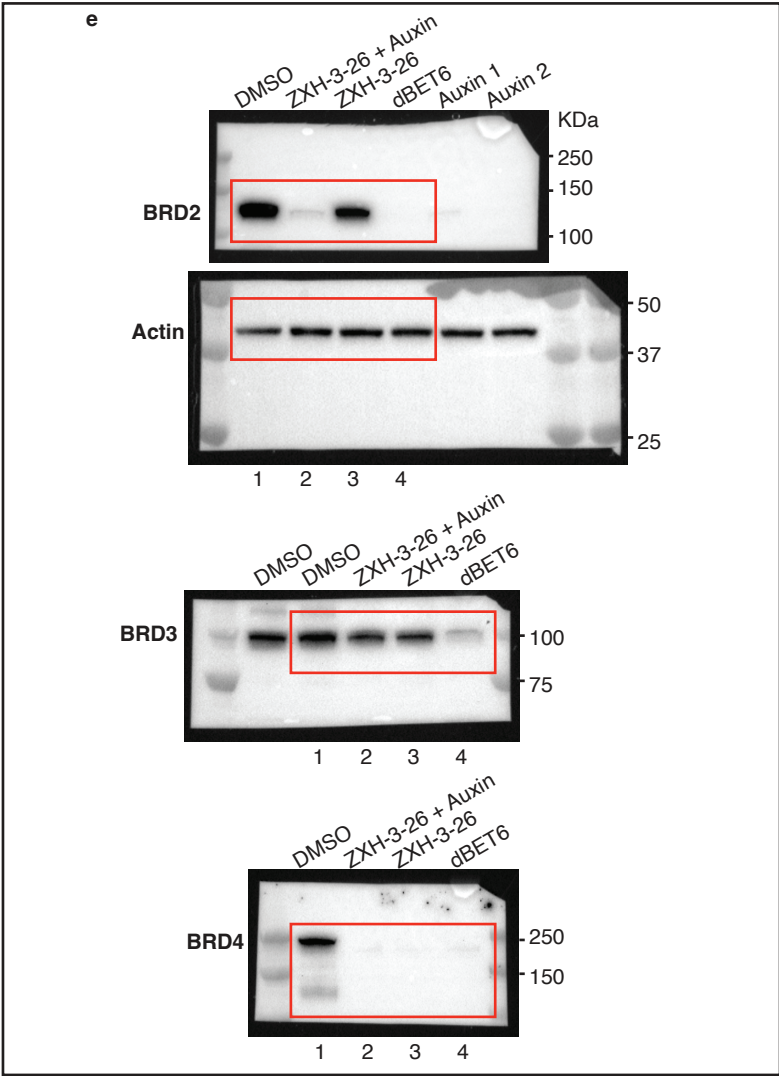

Supplement: Supplementary file 8 — Unprocessed western blots. [file 41588_2026_2533_MOESM8_ESM.pdf]

Extended Data Fig. 1

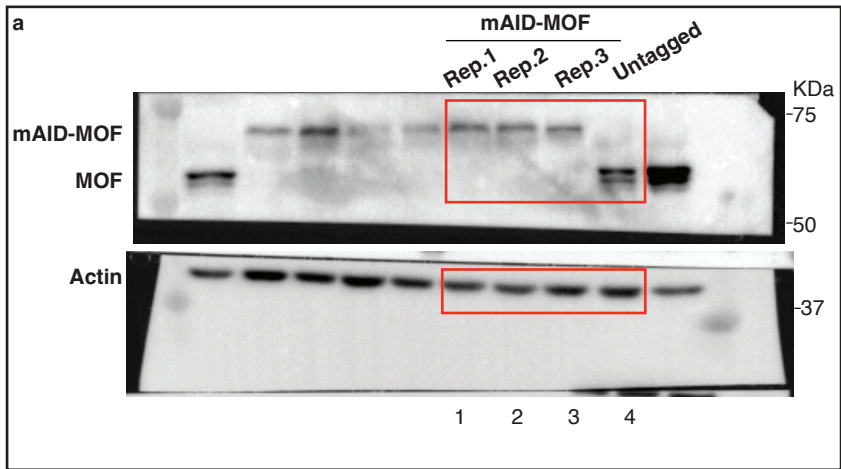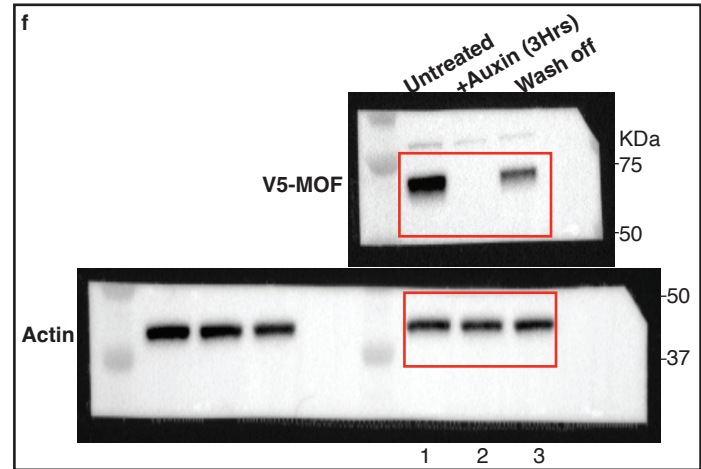

Supplement: Supplementary file 14 — Unprocessed western blots. [file 41588_2026_2533_MOESM14_ESM.pdf]

Extended Data Fig. 2

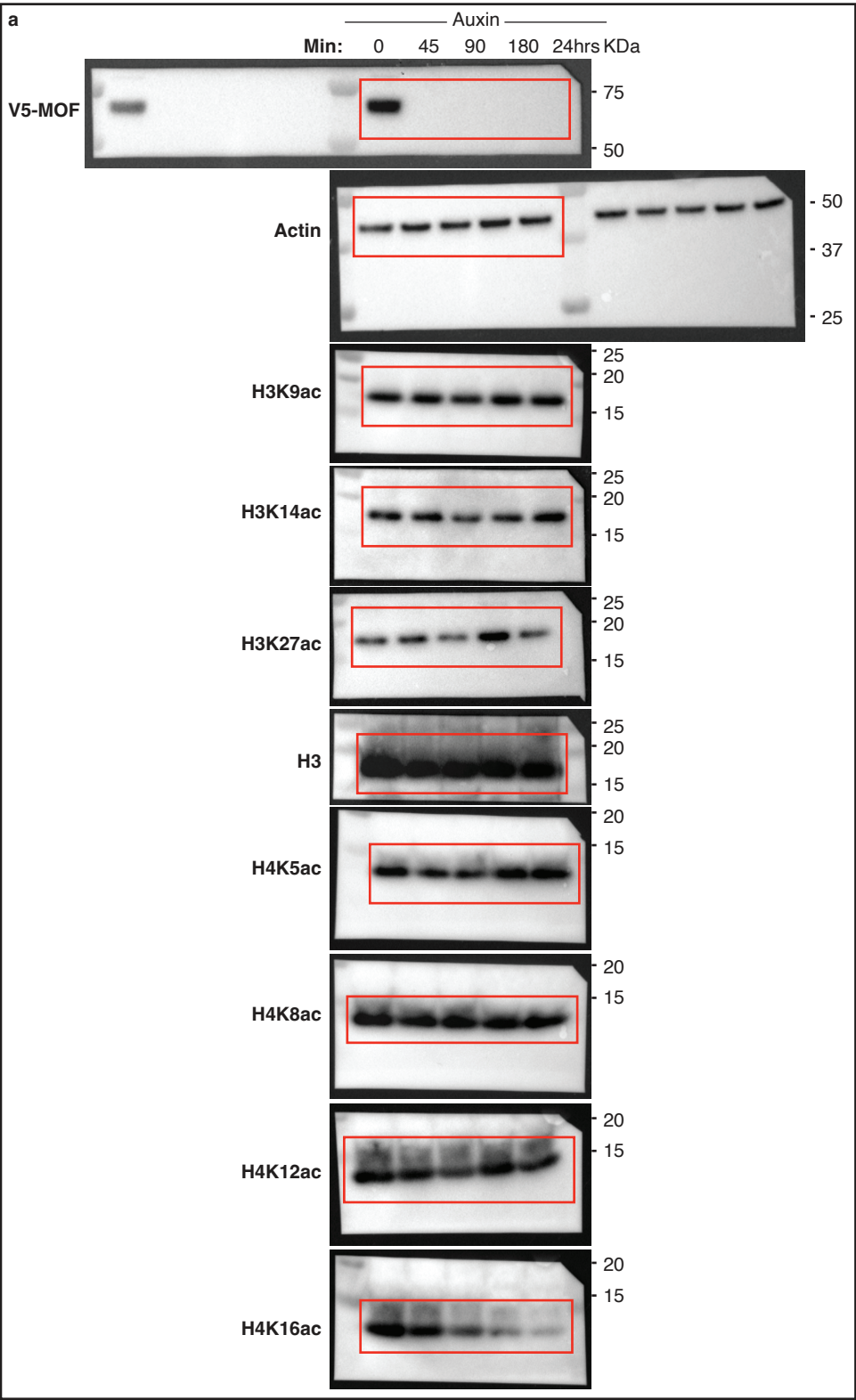

Supplement: Supplementary file 16 — Unprocessed western blots. [file 41588_2026_2533_MOESM16_ESM.pdf]

Extended Data Fig. 3

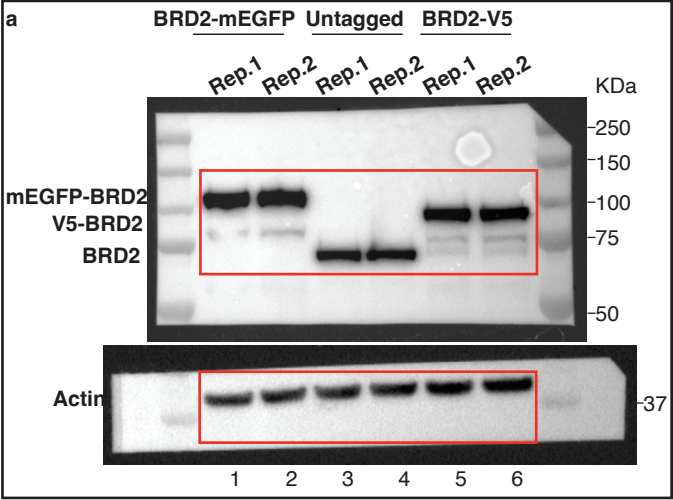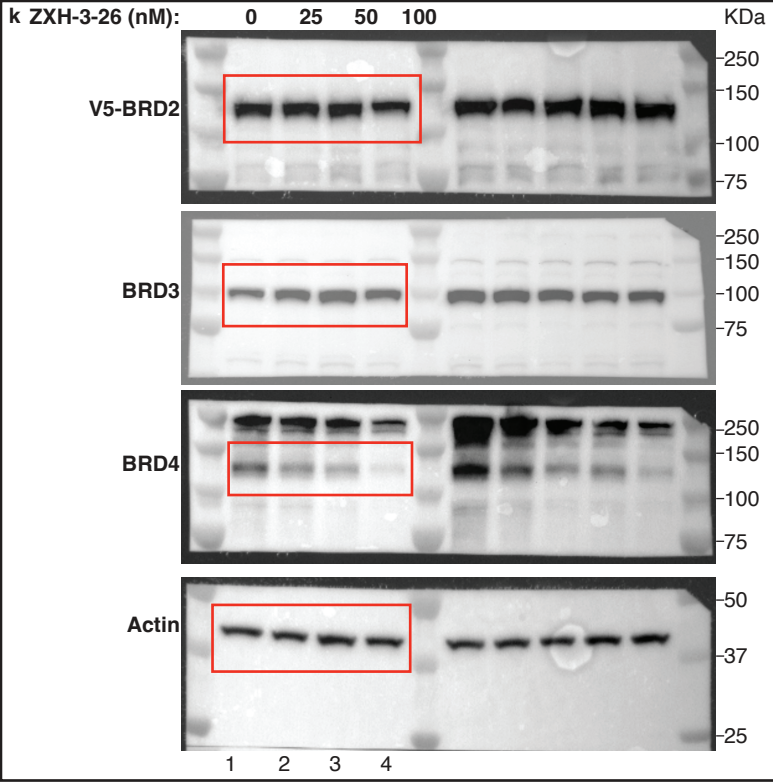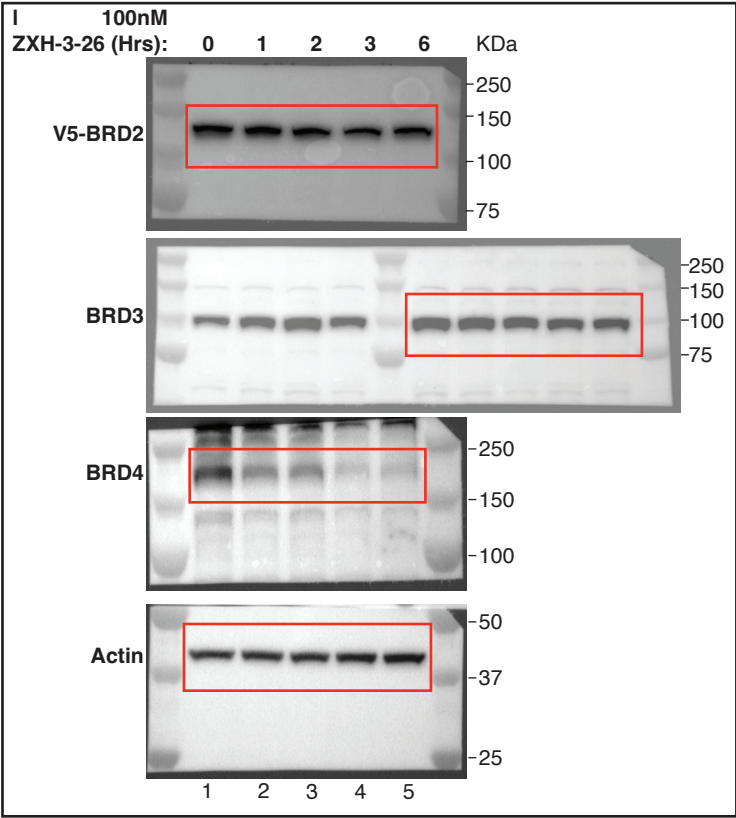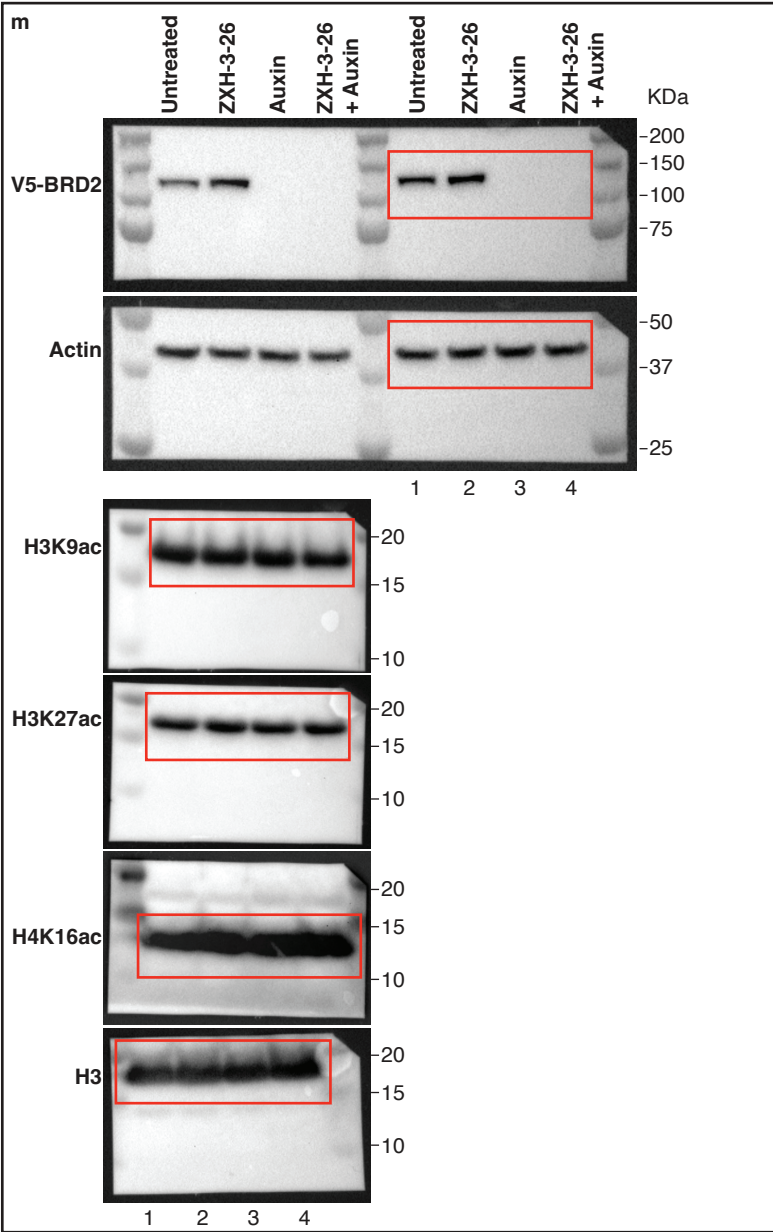

Supplement: Supplementary file 17 — Unprocessed western blots. [file 41588_2026_2533_MOESM17_ESM.pdf]

Extended Data Fig. 6

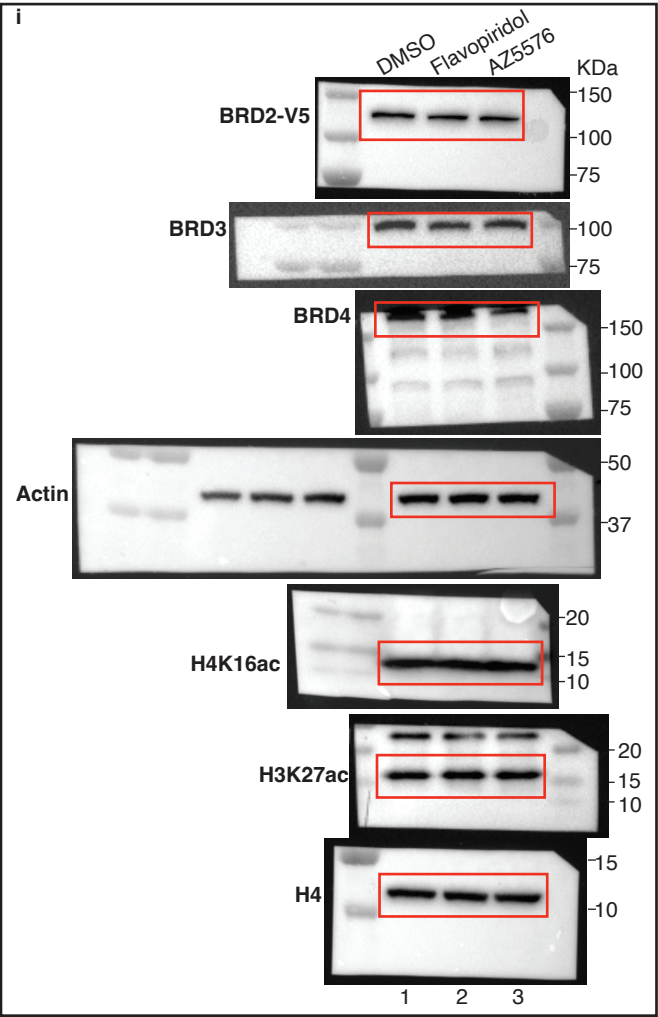

Supplement: Supplementary file 20 — Unprocessed western blots. [file 41588_2026_2533_MOESM20_ESM.pdf]

Extended Data Fig. 7

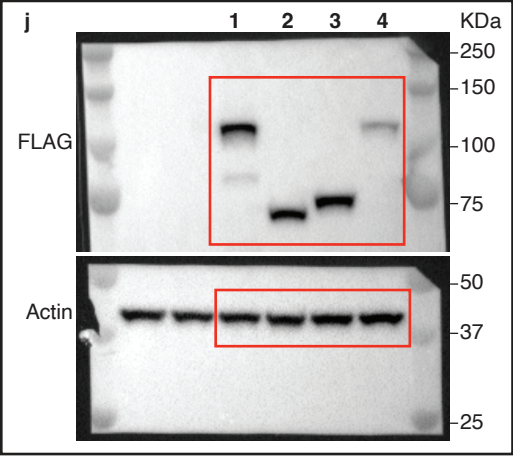

Supplement: Supplementary file 22 — Unprocessed western blots. [file 41588_2026_2533_MOESM22_ESM.pdf]

Extended Data Fig. 8

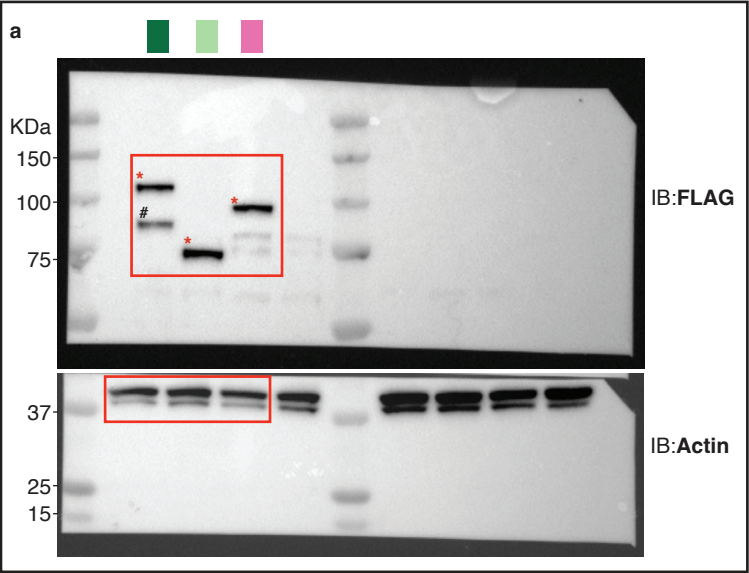

Supplement: Supplementary file 24 — Unprocessed western blots. [file 41588_2026_2533_MOESM24_ESM.pdf]
